# Targeting the Tie2–α_v_β_3_ integrin axis with bi-specific reagents for the inhibition of angiogenesis

**DOI:** 10.1186/s12915-018-0557-9

**Published:** 2018-08-17

**Authors:** Tomer Shlamkovich, Lidan Aharon, Dana Koslawsky, Yulia Einav, Niv Papo

**Affiliations:** 1 Department of Biotechnology Engineering and the National Institute of Biotechnology in the Negev, Ben-Gurion University of the Negev, P.O. Box 653, 84105 Beer-Sheva, Israel; 2 Faculty of Engineering, Holon Institute of Technology, Holon, Israel

**Keywords:** Angiogenesis, Bi-specific proteins, Directed evolution, Integrins, Protein engineering, Receptor tyrosine kinases

## Abstract

**Background:**

Increased activity of the receptor tyrosine kinase Tie2 has been implicated in the promotion of pathological angiogenesis. This activity is mainly mediated through angiopoietin (Ang)1- and Ang2-dependent activation of integrins by Tie2, rendering the Ang/Tie2/integrin axis an attractive putative target for cancer therapeutics.

**Results:**

To target this axis, we developed single domain, non-immunoglobulin high-affinity bi-specific protein inhibitors against both Tie2 and α_v_β_3_ integrin. We have previously engineered the Ang2-binding domain of Tie2 (Ang2-BD) as a Tie2 inhibitor. Here, we engineered an exposed loop in Ang2-BD to generate variants that include an integrin-binding Arg–Gly–Asp (RGD) motif and used flow cytometry screening of a yeast-displayed Ang2-BD RGD loop library to identify the integrin antagonists. The bi-specific antagonists targeting both Tie2 and α_v_β_3_ integrin inhibited adhesion and proliferation of endothelial cells cultured together with the α_v_β_3_ integrin ligand vitronectin, as well as endothelial cell invasion and tube formation. The bi-specific reagents inhibited downstream signaling by Tie2 intracellularly in response to its agonist Ang1 more effectively than the wild-type Ang2 BD that binds Tie2 alone.

**Conclusions:**

Collectively, this study—the first to describe inhibitors targeting all the known functions resulting from Tie2/integrin α_v_β_3_ cross-talk—has created new tools for studying Tie2- and integrin α_v_β_3_-dependent molecular pathways and provides the basis for the rational and combinatorial engineering of ligand–Tie2 and ligand–integrin α_v_β_3_ receptor interactions. Given the roles of these pathways in cancer angiogenesis and metastasis, this proof of principle study paves the route to create novel Tie2/integrin α_v_β_3_-targeting proteins for clinical use as imaging and therapeutic agents.

**Electronic supplementary material:**

The online version of this article (10.1186/s12915-018-0557-9) contains supplementary material, which is available to authorized users.

## Background

Angiogenesis, whether involving the normal or pathological behavior of vascular endothelial cells [1–6], is controlled by a balance of pro- and anti-angiogenic effectors in different pathways. In malignancy, combinations of cross-interacting pro-angiogenic signals [7, 8] activate endothelial cells attracted to the tumor microenvironment, thereby driving vascular growth [9, 10]. It has previously been suggested that inhibiting angiogenesis by targeting the regulation and cross-interaction of such signals could form the basis of efforts aimed at engineering cancer therapeutics [2, 11, 12]. Nonetheless, despite some progress in the field, anti-angiogenic therapeutic approaches targeting single components of cross-interacting signaling pathways have proved to have only limited clinical benefit, largely due to rapidly acquired resistance that enables endothelial cells in the tumor microenvironment to activate compensatory proliferative pathways [11, 13–15]. The current approach to dealing with this problem lies in cocktail therapeutics, many of which have already been introduced into clinical practice, albeit not always with the success predicted by preclinical trials [16–20].

Given the complexity and redundancy of angiogenic signaling pathways and their cross-interactions, the design of multi-component protein therapeutics, particularly multi-domain but also single domain therapeutics, that are able to perturb parallel nodes of critical angiogenesis-associated networks, has attracted considerable attention as a promising avenue to combat drug resistance in different cancers [21–23]. This concept has been applied, for example, to the development of antibody-based bi-specific inhibitors to vascular endothelial growth factor receptor-2 (VEGFR2)-α_v_β_3_ integrin and VEGFA-angiopoietin-2 (Ang2) [24–30]. These inhibitors are based on the key role of integrins as transmembrane linkers connecting their extracellular ligands with the cytoskeleton. This role allows integrins to influence cell migration during angiogenesis and to control the proliferation of vascular endothelial cells [31]. Although crosstalk between integrins and growth factor signaling pathways has been investigated in depth, such crosstalk for the Tie2 receptor tyrosine kinase (RTK)-integrin system has only recently been demonstrated, as has its major role in mediating angiogenesis [32, 33].

At the beginning of this decade, the Tie2–α_5_β_1_ integrin axis was first identified as a common module in angiogenesis [32, 33], although more recently another critical player in angiogenesis, namely α_v_β_3_ integrin, was identified as being part of this axis [34, 35]. It was also shown that Tie2 cross-interacts with α_v_β_3_ integrin, with putative pathobiological roles for the Tie2–α_v_β_3_ integrin axis in a diverse array of cancers having been suggested [35]. Like Tie2, α_v_β_3_ integrin is highly expressed on activated endothelial cells in the tumor neovasculature but only weakly expressed in resting endothelial cells and in most normal tissues and organs [36–38]. In the current study, we developed and evaluated the therapeutic potential of targeting the newly identified Tie2–α_v_β_3_ axis with the novel single domain, non-immunoglobulin bi-specific protein inhibitors against both Tie2 and α_v_β_3_ integrin.

Defining the mechanism of Tie2 involvement in endothelial cell proliferation, invasion, and angiogenesis is an evolving field. It has been shown that Tie2 triggers tumor-associated endothelial cell progression in the cancer microenvironment and significantly enhances the angiogenic and invasive potential of endothelial cells in vitro [39–41]. Accordingly, Tie2 suppression by RNA interference markedly reduced endothelial cell growth, proliferation, and invasive potential [42]. The pro-angiogenic and invasive potentials of Tie2 have also been demonstrated in in vivo models of angiogenesis [43]. For example, it is known that the ectopic expression of Tie2 correlates well with increased endothelial cell proliferation and migration in vivo [44, 45].

The exact mechanism downstream of Tie2 activation, specifically its interaction with integrins and ECM components, remains largely elusive. Research on the interactions between Tie2 and integrins has shown that Tie2 readily associates with α_v_β_3_ integrin through their respective ectodomains [35]. It was further demonstrated that the Tie2 agonistic ligand Ang1 [46–48], but not Ang2, an angiopoietin family member with Tie2 antagonistic activity [49], can independently associate with α_v_β_3_ integrin, resulting in increased motility of endothelial cells [32, 35]. These Ang1–integrin and Tie2–integrin interactions are independent of the Arg–Gly–Asp (RGD) tripeptide motif that facilitates interactions of integrins with their natural extracellular matrix (ECM) ligands, including fibronectin, vitronectin, fibrinogen, and osteopontin [37, 50]. Of the 24 integrin αβ heterodimers, 8 are known to recognize the RGD sequence in their ligands. Determination of the crystal structures of α_v_β_3_, α_IIb_β_3_, and α_5_β_1_ integrins interacting with soluble RGD-containing ligands has enabled elucidation of the mode of binding of the RGD motif to the integrin headpiece [51–53].

Exploiting current understanding of Tie2–Ang2-binding domain (Ang2-BD) interactions, our group has recently employed a combinatorial engineering approach to transform Ang2-BD into a highly potent Tie2 inhibitor with enhanced anti-angiogenic and anti-invasive activities on endothelial cells [54]. Here, we extended the work by developing Ang2-BD-based bi-specific inhibitors that simultaneously target α_v_β_3_ integrin and their immediate in vivo target Tie2. The dual Tie2 inhibitors and α_v_β_3_ integrin antagonists were generated from affinity screens of an Ang2-BD_RGD_ loop library. The affinity-matured bi-specific proteins were expressed as soluble proteins and were shown to bind simultaneously, with diverse affinities, to both Tie2 and α_v_β_3_ integrin, while presenting high inhibitory and antagonistic activities in cells*.* Furthermore, the bi-specific protein inhibitors displayed superior therapeutic potential, as compared to Tie2 or α_v_β_3_ integrin mono-treatments, as reflected in endothelial cell adhesion, and Tie2, Akt, and FAK phosphorylation; Tie2 localization at cell-cell junctions; tube formation; and endothelial cell proliferation and invasiveness. The results provide further evidence of Tie2 crosstalk with α_v_β_3_ integrins and suggest putative pathobiological roles for the Tie2–α_v_β_3_ integrin axis in angiogenesis. Our findings, moreover, support the premise that the Tie2–α_v_β_3_ integrin axis offers an attractive target for the development of novel anti-angiogenic therapeutics.

## Results

### Construction and screening of a bi-specific Ang2-BD library that binds both Tie2 and α_v_β_3_ integrin

**Fig. 1:**
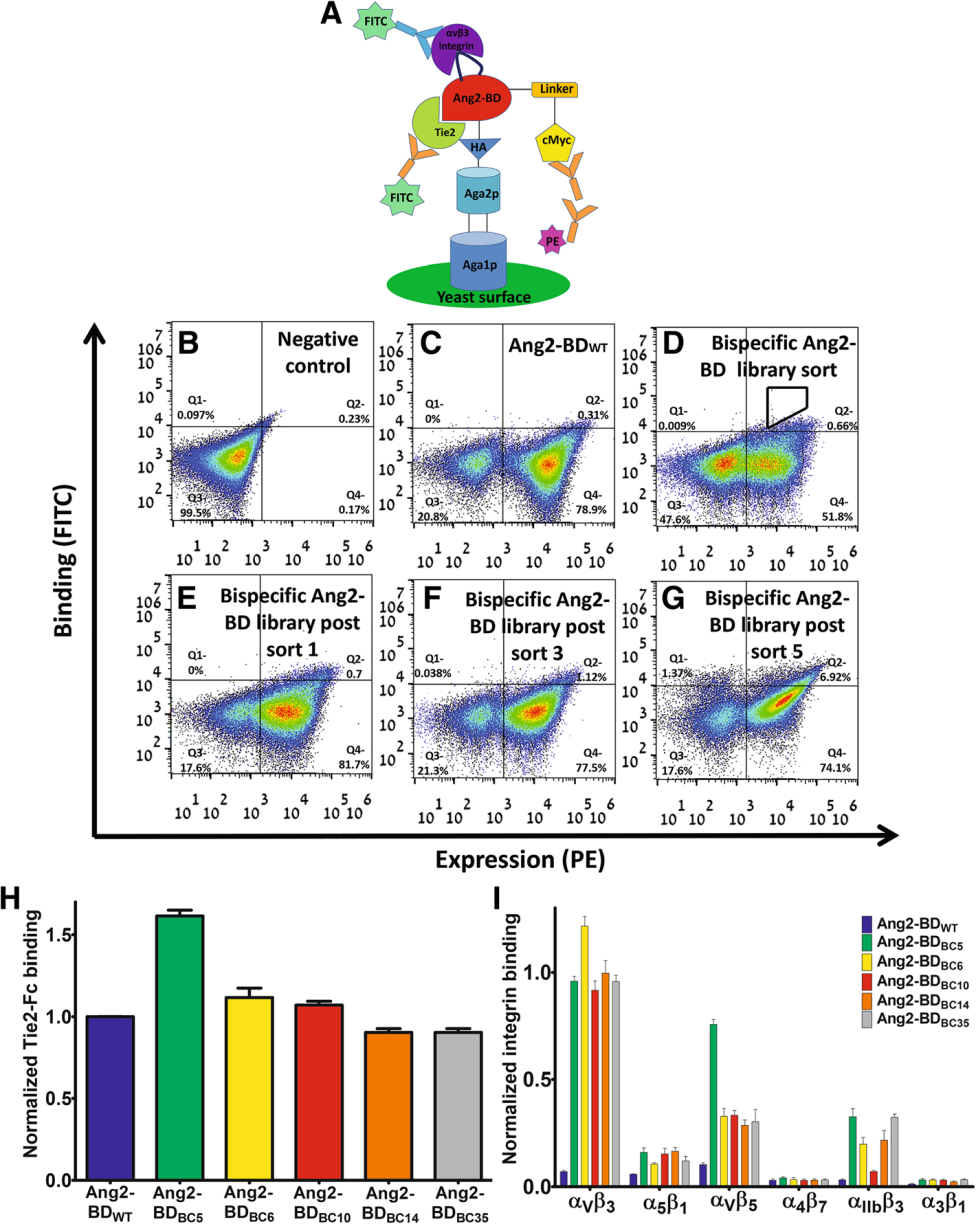
Affinity maturation of the Ang2-BD_RGD_-based library bi-specific for α_v_β_3_ integrin and Tie2-Fc. **a** Ang2-BD was presented on the yeast cell surface as a fusion with agglutinin proteins. Display levels were detected using primary antibodies against the C-terminal cMyc tag (chicken anti-cMyc antibodies) and phycoerythrin (PE)-conjugated anti-chicken antibodies. Binding to Tie2-Fc was determined using fluorescein isothiocyanate (FITC)-conjugated anti-human Fc antibodies. Binding to α_v_β_3_ integrin was determined using FITC-labeled mouse anti-α_v_ integrin antibodies. **b**–**g** FACS analysis of the binding of the bi-specific Ang2-BD-based library to α_v_β_3_ integrin in different screening steps. Quadrant gate statistics are indicated in each panel **b** negative control. **c** Ang2-BD_WT_ expression and α_v_β_3_ integrin binding (10 nM). **d** Expression of the bi-specific Ang2-BD_RGD_-based library and α_v_β_3_ integrin binding (10 nM) at pre-sorting and **e**–**g** expression of the bi-specific Ang2-BD-based library and α_v_β_3_ integrin binding (10 nM) after sorts 1, 3, and 5, respectively. **h** Binding of isolated yeast-displayed bi-specific Ang2-BD_RGD_ clones to Tie2 (20 nM). Data were normalized to the yeast surface expression levels of each clone and Tie2 binding of Ang2-BD_WT_. **i** Binding of isolated yeast-displayed bi-specific Ang2-BD_RGD_ clones to α_v_β_3_, α_5_β_1_, α_v_β_5_, α_4_β_7_, α_IIb_β_3_, and α_3_β_1_ integrins (50 nM). Data were normalized to the yeast surface expression level of each clone. Data shown represent the average of triplicates from independent experiments, and error bars represent the standard error of the mean

To develop bi-specific Ang2-BD protein antagonists, we generated a YSD library in which one of the Ang2-BD-exposed loops (residues 301–308) was replaced by the RGD motif flanked by three random amino acids on each side. For library screening, the Ang2-BD library was cloned into a YSD plasmid and presented on the yeast cell surface, and binding to Tie2 and α_v_β_3_ integrin was detected by FACS (after staining with fluorescently-labeled antibodies, as opposed to non-stained controls). The position of the loop library was chosen such that it could bind α_v_β_3_ integrin without disrupting the binding of the resulting Ang2-BD_RGD_ protein variant to its native receptor, Tie2 (Fig. 1a). The bi-specific Ang2-BD_RGD_-based library was subjected to five rounds of high-throughput flow cytometry sorting using decreasing concentrations of α_v_β_3_ integrin (Fig. 1d–g). Sorts 2–5 were performed using the gate shown in Fig. 1d. As expected, the wild-type protein Ang2-BD_WT_ did not bind to α_v_β_3_ integrin (Fig. 1c).

### Isolation of bi-specific clones that bind to both Tie2 and α_v_β_3_ integrin

Sequencing analysis of the bi-specific clones isolated from the fifth sort of the Ang2-BD_RGD_-based library verified that the integrin-binding loop did indeed contain the RGD motif flanked by random amino acids and that the RGD motif was located in the middle of the loop sequence (Table 1). Importantly, the isolated yeast-displayed variants maintained their binding affinity towards Tie2, despite the insertion of the RGD epitope into Ang2-BD (Fig. 1h). The isolated clones were shown to bind α_v_β_3_ integrin, whereas Ang2-BD_WT_ did not (Fig. 1i). To determine the specificity of α_v_β_3_ integrin binding and to establish that the bi-specific variants did not interfere with essential biological functions, it was necessary to first investigate the ability of the yeast-displayed variants to bind to other integrins—both those implicated in angiogenesis and those with biological functions not associated with angiogenesis, namely α_5_β_1_ and α_v_β_5_ integrins (involved in pathological angiogenesis) [37], α_3_β_1_ integrin (promotes tumor cell adhesion migration and invasion) [65], α_IIb_β_3_ integrin (involved in platelet aggregation) [66], and α_4_β_7_ integrin (functions in leukocyte recruitment) [67]. The binding results showed that the isolated clones all bound strongly to α_v_β_3_ integrin yet only bound weakly to the other two angiogenesis-promoting integrins (α_5_β_1_ and α_v_β_5_) and to α_IIb_β_3_ integrin, and not at all to α_3_β_1_ and α_4_β_7_ integrins (Fig. 1i).

**Table 1:** Sequencing analysis of isolated clones from the bi-specific Ang2-BD_RGD_ library

Isolated Ang2-BD_RGD_ clone	Loop sequence
Ang2-BD_BC5_	NTCRGDCLP
Ang2-BD_BC6_	REGRGDNVD
Ang2-BD_BC10_	YPGRGDNPD
Ang2-BD_BC14_	GRRRGDMPD
Ang2-BD_BC35_	YEPRGDNPS
Fibronectin	VTGRGDSPA
Vitronectin	QVTRGDVFT

### Purification and evaluation of soluble Ang2-BD bi-specific proteins

**Fig. 2:**
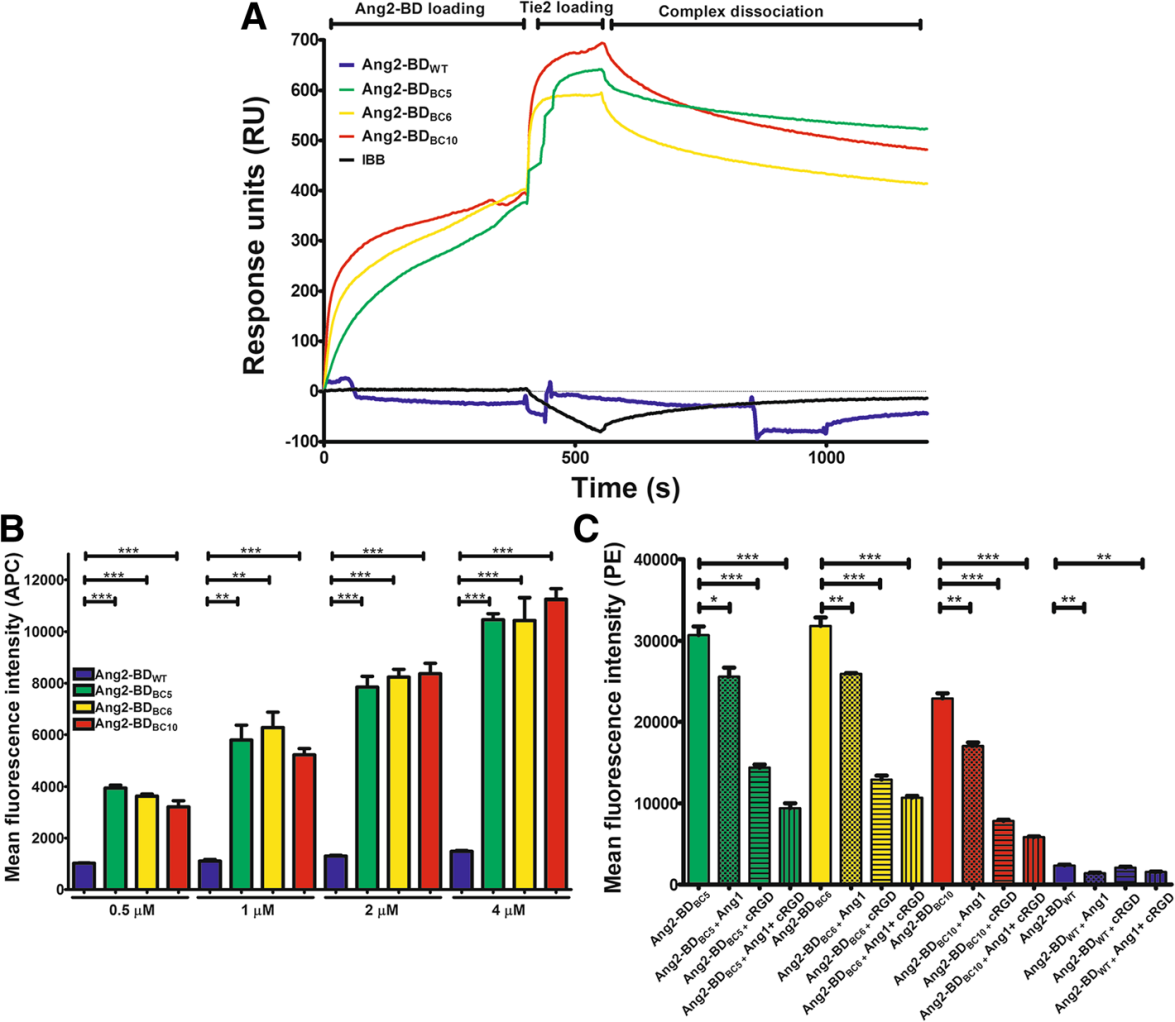
Binding of Ang2-BD bi-specific variants to recombinant and cell-expressed human Tie2 and α_v_β_3_ integrin. **a** Representative SPR sensorgram of Ang2-BD bi-specific variants (400 nM) binding to both α_v_β_3_ integrin (immobilized) and soluble Tie2 (400 nM). Ang2-BD variants **[**Ang2-BD_WT_ (blue), Ang2-BD_BC5_ (green), Ang2-BD_BC6_ (yellow), or Ang2-BD_BC10_ (red)] were allowed to flow for 400 s followed by an additional 150 s flow of Tie2. Injection of running buffer followed by rhTie2 in IBB served as negative control (black). Injection steps are indicated on the sensorgrams. **b** Binding of Ang2-BD bi-specific variants to TIME cells. 1 × 10^5^ cells were incubated with Ang2-BD_WT_ (blue), Ang2-BD_BC5_ (green), Ang2-BD_BC6_ (yellow), or Ang2-BD_BC10_ (red) for 2 h at 4 °C with gentle agitation. Mean fluorescence values were determined by flow cytometry using fluorescently labeled antibodies against a FLAG epitope tag. “*” indicates a *P* value < 0.05 by one-way ANOVA upon comparing results between Ang2-BD variants at the same concentration. **c** Competitive binding of 1 μM Ang2-BD_WT_ (blue), Ang2-BD_BC5_ (green), Ang2-BD_BC6_ (yellow), or Ang2-BD_BC10_ (red), alone or with a combination of 1000 ng/ml of FL-Ang1 (checkered bars), 10 μM cRGD (horizontally lined bars) and both 1000 ng/ml FL-Ang1 and 10 μM cRGD (vertically-lined bars). Mean fluorescence values were determined by flow cytometry using fluorescently labeled antibodies against a FLAG epitope tag. “*” indicates a *P* value < 0.05 by one-way ANOVA upon comparing results between Ang2-BD variants alone and with FL-Ang1 and cRGD competitors. Data shown represent the average of triplicates from independent experiments, and error bars represent the standard error of the mean

Based on the binding affinity results for both Tie2 and α_v_β_3_ integrin binding obtained from YSD, three Ang2-BD bi-specific clones (BC), designated Ang2-BD_BC5_, Ang2-BD_BC6_, and Ang2-BD_BC10_ and produced as soluble proteins (Additional file 1: Figure S1), were chosen for further experimentation for the following reasons: these bi-specific variants retained their ability to bind to Tie2, as shown by surface plasmon resonance (SPR) (Additional file 1: Figure S2A), with the *K*_*D*_ values of the three variants lying in the range of 0.95–1.36 μM vs 0.66 μM for Ang2-BD_WT_ (Table 2). Furthermore, these bi-specific variants also bound to α_v_β_3_ integrin (Additional file 1: Figure S2B), while Ang2-BD_WT_ did not. The rate constants for the binding kinetics of the bi-specific variants to α_v_β_3_ integrin (*K*_D_ 2.97–14.9 nM Table 2) demonstrated that the engineered RGD loop grafted into Ang2-BD produced a new binding epitope that facilitated strong affinity to α_v_β_3_ integrin without disrupting its original functionality of binding to Tie2 (as can be seen from the comparable affinity values for the clones and Ang2-BD_WT_). These findings were supported by a dual SPR binding experiment that showed that all the Ang2-BD bi-specific variants simultaneously bound Tie2 and α_v_β_3_ integrin (Fig. 2a), whereas no direct interaction between immobilized α_v_β_3_ integrin and soluble Tie2 was seen. To complement the YSD experiments in which the yeast-displayed Ang2-BD bi-specific variants were tested for their binding to different types of soluble integrin, binding of the soluble bi-specific variants to different soluble integrins was tested. The SPR results demonstrated that the Ang2-BD bi-specific variants could bind other integrins that are overexpressed in cancer and in the tumor vasculature, such as α_v_β_5_ and α_5_β_1_ integrins, but do not bind to other integrins (such as α_3_β_1_, α_IIb_β_3_, and α_4_β_7_) that are less dominantly expressed in cancer and the tumor vasculature. The Ang2-BD bi-specific variants were also found to bind to other α_v_ integrins, such as α_v_β_1_, α_v_β_6_, and α_v_β_8_ integrins, albeit with weaker affinity than to α_v_β_3_ integrin (Additional file 1: Figure S2C–D). Evaluation of the binding of Ang2-BD_BC5_ to other α_v_ integrins yielded in *K*_*D*_ values of 550, 65, 364, and 138 nM for α_v_β_1_, α_v_β_5_, α_v_β_6_, and α_v_β_8_ integrins, respectively (Table 3), values 4 to 37-fold higher than for α_v_β_3_ integrin (14.9 nM).

**Table 2:** Equilibrium binding affinities and kinetic rate constants for binding of Ang2-BD variants to immobilized Tie2 and α_v_β_3_ integrin

Variant	SPR (immobilized Tie2): steady state	SPR (immobilized α_v_β_3_ integrin): 1:1 Langmuir binding model		
	*K*_*D*_ ± sem, μM	*K*_*D*_ ± sem, nM	*K*_on_ ± sem, (M^−1^ s^−1^) × 10^4^	*K*_off_ ± sem, (s^−1^) × 10^−4^
Ang2-BD_WT_	0.66 ± 0.04	*	*	*
Ang2-BD_BC5_	1.36 ± 0.68	14.9 ± 1.14	2.99 ± 0.01	4.44 ± 0.03
Ang2-BD_BC6_	1.26 ± 0.18	2.97 ± 0.34	7.94 ± 0.02	2.36 ± 0.01
Ang2-BD_BC10_	0.95 ± 0.1	4.17 ± 0.54	10.3 ± 0.01	4.29 ± 0.02

Values in the table are means ± SEM. SPR sensorgram curves were fitted to a steady state model (for Tie2 binding) and a 1:1 Langmuir binding model (for α_v_β_3_ integrin binding)

*Binding was not observed for Ang2-BD variants in the 2 μM–125 nM range

**Table 3:** Binding affinities and kinetic rate constants for binding of Ang2-BD _BC5_ to immobilized integrins

Integrin	*K*_*D*_ ± sem, nM	*K*_on1_ ± sem (M^−1^ s^−1^) × 10^4^	*K*_off1_ ± sem (s^−1^) × 10^−3^	*K*_on2_ ± sem (s^−1^) × 10^−3^	*K*_off2_ ± sem (s^−1^) × 10^−3^
α_v_β_1_	550 ± 90.1	1.14 ± 0.01	6.29 ± 0.17	3.04 ± 0.12	1.97 ± 0.03
α_v_β_5_	65 ± 21.8	3.98 ± 0.03	2.59 ± 0.12	5.44 ± 0.21	0.35 ± 0.01
α_v_β_6_	364 ± 70.8	2.29 ± 0.05	8.35 ± 0.45	3.2 ± 0.17	1.06 ± 0.03
α_v_β_8_	138 ± 50.3	3.66 ± 0.03	5.05 ± 0.13	2.4 ± 0.12	2.15 ± 0.05

Values in the table are means ± SEM. SPR sensorgram curves were fit to a two-state binding model

### Binding of Ang2-BD bi-specific variants to cell-expressed Tie2 and integrin

The binding capabilities of Ang2-BD bi-specific variants to cell-expressed Tie2 and integrin were evaluated in TIME cells, which express Tie2 and α_v_β_3_ integrin on their surface (Additional file 1: Figure S3). All of the Ang2-BD bi-specific proteins exhibited strong binding (relative to Ang2-BD_WT_) to TIME cells in a dose-response manner (Fig. 2b). To confirm that the Ang2-BD bi-specific variants specifically bound to both Tie2 and α_v_β_3_ integrin, we employed a competitive binding assay in which full-length human Ang1 (FL-Ang1) and/or a tenfold molar excess of the cRGD peptide (vs Ang2-BD proteins) competed with the bi-specific engineered proteins for binding to Tie2 and α_v_β_3_ integrin, respectively. Upon competition with FL-Ang1 for binding to Tie2, decreases in binding for Ang2-BD_BC5_, Ang2-BD_BC6_, and Ang2-BD_BC10_ were 17%, 19%, and 26%, respectively. Upon competition with cRGD for binding to α_v_β_3_ integrin, the respective decreases in binding were 53%, 60%, and 66%. When both FL-Ang1 and cRGD were added together as inhibitors, cell binding to Ang2-BD_BC5_, Ang2-BD_BC6_, and Ang2-BD_BC10_ decreased by 70%, 66%, and 75%, respectively. These results demonstrate that the Ang2-BD bi-specific variants did indeed bind to both targets (Fig. 2c).

### Docking modeling and simulation of the Ang2-BD_BC5_–α_v_β_3_ integrin complex

To visualize and characterize the binding of the Ang2 mutants to α_v_β_3_ integrin, we prepared a docking model of the Ang2-BD_BC5_–α_v_β_3_ integrin complex as a representative model for the Ang2 mutants. The structure was simulated using a molecular dynamics (MD) method for 10 ns until a stable interface between Ang2-BD_BC5_ and α_v_β_3_ integrin was reached (Additional file 1: Figure S4). To characterize specific interactions between Ang2-BD_BC5_ and α_v_β_3_ integrin, the Gromacs package was used to measure minimal distances and Coulomb and Lennard–Jones potentials of the simulated Ang2-BD_BC5_–α_v_β_3_ integrin complex. Additional file 1: Figure S5 presents the most important bonds between the RGD sequence and the β_3_ subunit, while Additional file 1: Table S1 summarizes these interactions. In the β_3_ subunit, the fully charged side chains of Arg304 and Asp217 constitute a strong salt bridge. At the same time, the backbone oxygen atom of Arg304 comes into a contact with a positively charged amine group of Lys253, creating a strong polar interaction. The carbonyl oxygen of Gly305 is inserted into the positively charged environment of the Lys253 and Asn215 side chain amine groups. Asp306 comes into contact with two side chain amine groups of the β_3_ subunit, Asn215, and Tyr122. Although the side chain of Asp306 points towards the α_v_ subunit, its contact with the α_v_ residues is intermittent and less significant than that with β_3_, and thus is not shown here. The RGD flanking residues of Ang2-BD_BC5_ make several weaker and less significant contributions to α_v_β_3_ integrin binding (data not shown).

**Fig. 3:**
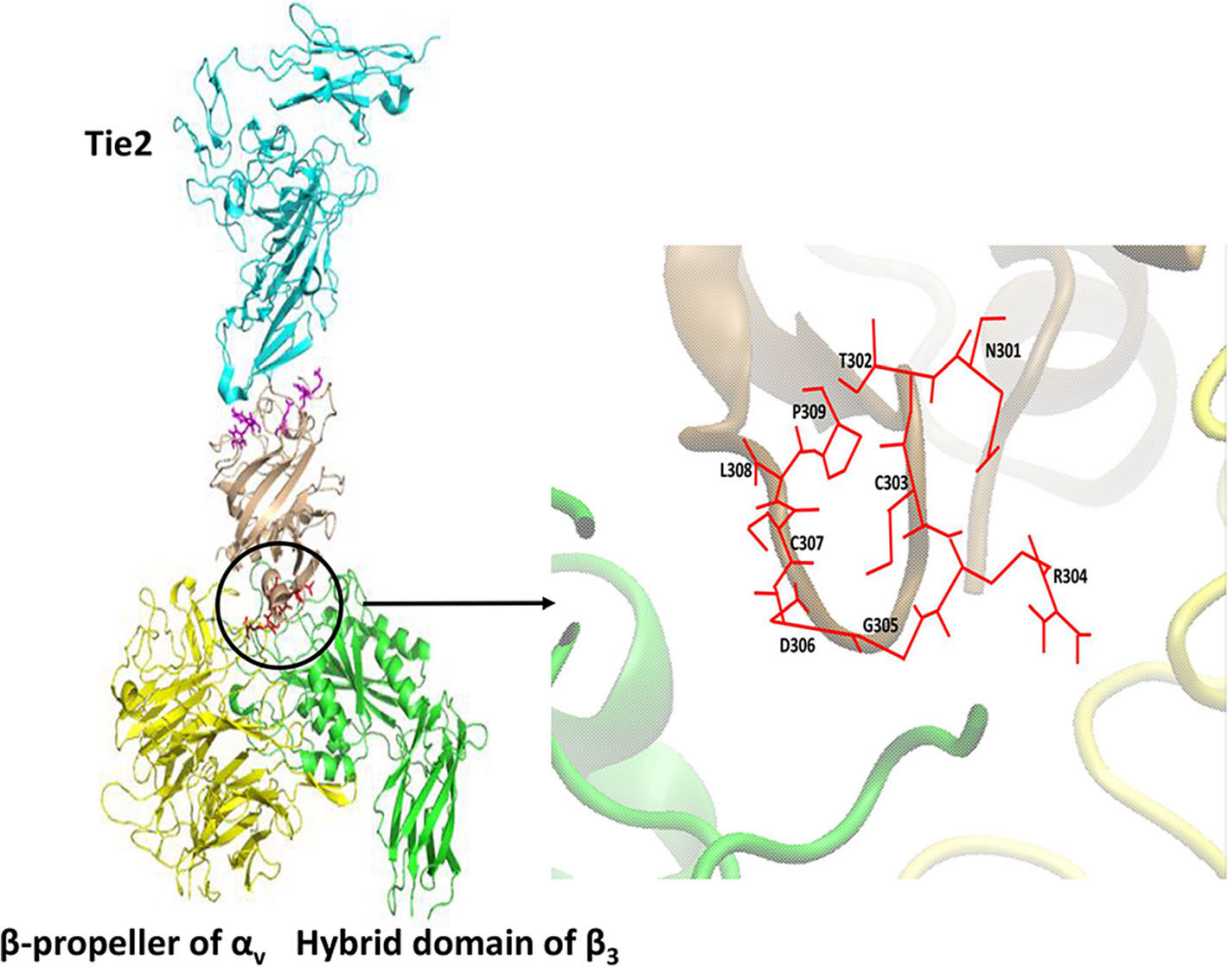
Structure of the Tie2–Ang2-BD_BC5_–α_v_β_3_ complex. The Tie2 structure was aligned into the last frame of the Ang2-BD_BC5_–integrin α_v_β_3_ MD simulation. Tie2 is shown in cyan, Ang2-BD_BC5_ in beige, α_v_ in yellow, and β_3_ in green. Tie2-binding residues of Ang2 are colored magenta and NTCRGDCLP mutant residues in red. A zoom-in of the binding interface between Ang2-BD_BC5_ and α_v_β_3_ integrin is also shown. On the right, an all-atom representation of the mutant residues of Ang2-BD_BC5_ is shown in stick form

The final structure aligned to the Tie2 receptor (Fig. 3) showed that Tie2 and the α_v_β_3_ integrin-binding domains are located on the opposite sides of Ang2-BD_BC5_ molecule, allowing simultaneous binding of the two receptors. The binding interface of the model of Ang2-BD_BC5_–α_v_β_3_ integrin complex is composed mainly of the RGD motif and its flanking residues from Ang2-BD_BC5_, together with both subunits of α_v_β_3_ integrin (Fig. 3). The mutant residues colored red in Fig. 3 protrude into the cleft between the α_v_ and the β_3_ integrin domains, with the side chain of Arg pointing towards the α_v_ subunit, Asp, and Gly making contact with the β_3_ subunit. MD simulations of the Ang2-BD_BC5_–α_v_β_3_ integrin complex demonstrated a possible mode of interaction between Ang2 mutants bearing the RGD sequence and α_v_β_3_ integrin. In the model, the RGD of Ang2-BD_BC5_ makes multiple contacts with its natural binding site on the top of the α_v_β_3_ integrin headpiece, which could cumulatively facilitate strong binding of the ligand to the integrin.

### Inhibition of integrin-mediated adhesion of TIME cells by Ang2-BD bi-specific variants

**Fig. 4:**
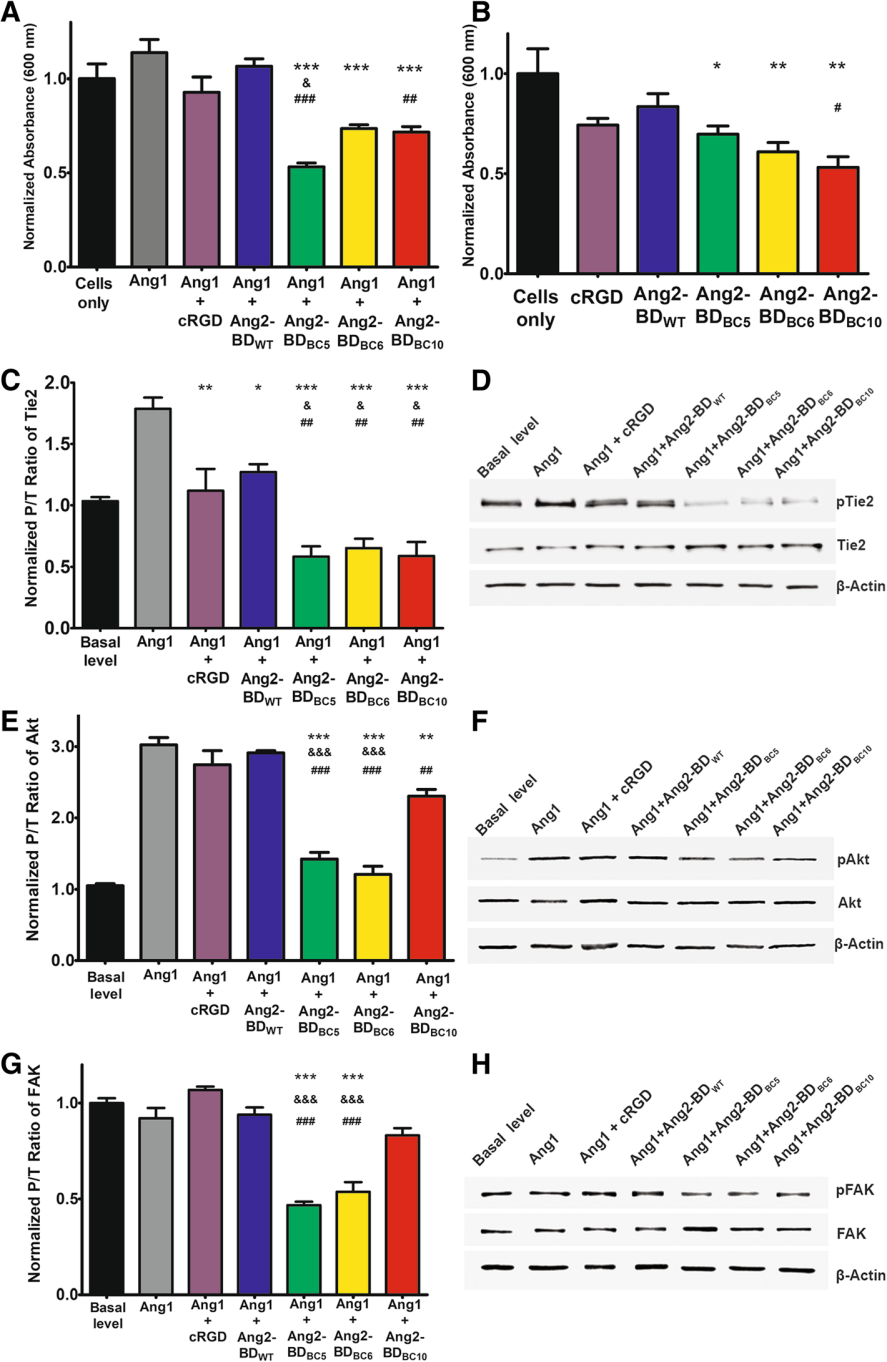
Inhibition of TIME cells adhesion and phosphorylation of Tie2, Akt, and FAK. **a** 5 × 10^4^ TIME cells were incubated alone (cells only; black), with 500 ng/ml of FL-Ang1 (gray), or with a combination of 500 ng/ml FL-Ang1 and 1 μM cRGD (purple), Ang2-BD_WT_ (blue), Ang2-BD_BC5_ (green), Ang2-BD_BC6_ (yellow), or Ang2-BD_BC10_ (red) for 2 h on vitronectin-coated 96-well plates. **b** 5 × 10^4^ TIME cells were incubated alone (cells only; black) or with 1 μM cRGD (purple), Ang2-BD_WT_ (blue), Ang2-BD_BC5_ (green), Ang2-BD_BC6_ (yellow), or Ang2-BD_BC10_ (red) for 2 h on vitronectin-coated 96-well plates. **c** For determining Tie2 phosphorylation, TIME cells were treated with control buffer (basal level; black), 500 ng/ml FL-Ang1 (gray), or a combination of 500 ng/ml FL-Ang1 with 1 μM cRGD (purple), Ang2-BD_WT_ (blue), Ang2-BD_BC5_ (green), Ang2-BD_BC6_ (yellow), or Ang2-BD_BC10_ (red) for 15 min on vitronectin-coated 12-well plates. **d** Cell lysates were analyzed by Western blot using antibodies against phosphorylated Tie2 (pTie2), Tie2, and β-actin. **e** Akt phosphorylation was determined as in **c** except that the cells were treated with a combination of 500 ng/ml FL-Ang1 with 0.5 μM of the above proteins and the incubation time was 30 min. **f** Cell lysates were analyzed by Western blot using antibodies against phosphorylated Akt (pAkt), Akt, and β-actin. **g** FAK phosphorylation was determined as in **c**. **h** Cell lysates were analyzed by Western blot using antibodies against phosphorylated FAK (pFAK), FAK, and β-actin. “*” indicates a *P* value < 0.05 upon comparing the results between the cells-only control (**b**) and the tested proteins. “*” indicates a *P* value < 0.05 upon comparing the results between FL-Ang1 alone (**a**, **c**, **e**, **h**) and a combination of FL-Ang1 with the tested proteins. “&” indicates a *P* value < 0.05 upon comparing the results between cRGD (**a**, **b**, **c**, **e**, **h**) and the tested proteins. “#” indicates a *P* value < 0.05 upon comparing the results between Ang2-BD_WT_ (**a**, **b**, **c**, **e**, **h**) and the tested proteins

An adhesion assay of TIME cells performed in vitronectin-coated plates demonstrated that the Ang2-BD bi-specific variants (1 μM) inhibited integrin-mediated adhesion in the presence of FL-Ang1. The adhesion capability of the TIME cells decreased by 53%, 35%, and 37% for cells treated with Ang2-BD_BC5_, Ang2-BD_BC6_, and Ang2-BD_BC10_, respectively, relative to the mild inhibition of 6% and 18% observed for the mono-specific Ang2-BD_WT_ protein and the cRGD (1 μM), respectively (Fig. 4a). When FL-Ang1 was not added, the adhesion capability of the TIME cells decreased by 30%, 39%, and 47% for cells treated with Ang2-BD_BC5_, Ang2-BD_BC6_, and Ang2-BD_BC10_, respectively, as opposed to the inhibition of 16% and 26% for the mono-specific Ang2-BD_WT_ protein and cRGD (1 μM), respectively (Fig. 4b). These findings indicate that FL-Ang1 enhanced the adhesion of the cells via the mediation of α_v_β_3_ integrin and its ligand, vitronectin, and hence imply that Ang1 plays a role in the Tie2–α_v_β_3_ integrin axis (compare Fig. 4a, b).

### Inhibition of Tie2, Akt, and FAK phosphorylation by Ang2-BD bi-specific variants in TIME cells

To test the ability of Ang2-BD bi-specific variants to inhibit Tie2, we employed a phosphorylation assay on TIME cells growing in vitronectin-coated plates. The assay is based on the induction of the phosphorylation of Tie2 and its downstream pathway PI3K/Akt by basal levels of endogenous Ang1, which, in turn, promotes endothelial cell migration and survival [68, 69, 70]. By enhancing such phosphorylation through the addition of soluble FL-Ang1 (500 ng/ml) to the cell culture [46], we tested the ability of the Ang2-BD bi-specific variants to compete with FL-Ang1 and thereby determine whether the specific effect of the variants on Tie2 phosphorylation is indeed mediated by FL-Ang1. The results demonstrated that the Ang2-BD bi-specific variants (1 μM) significantly inhibited Tie2 phosphorylation as induced by both endogenous and soluble FL-Ang1 and thus act as functional antagonists. The Ang2-BD bi-specific variants were more potent antagonists than Ang2-BD_WT_ and cRGD peptide (1 μM), as Tie2 phosphorylation intensity was decreased by 67%, 64%, and 67% for Ang2-BD_BC5_, Ang2-BD_BC6_, and Ang2-BD_BC10_, respectively, but only by 29% and 37% for Ang2-BD_WT_ and cRGD, respectively (Fig. 4c, d). To test the downstream signaling induced by Tie2 phosphorylation, we similarly evaluated the phosphorylation levels of Akt in TIME cells in vitronectin-coated plates. The results demonstrated that the Ang2-BD bi-specific variants (0.5 μM) significantly inhibited Akt phosphorylation induced by both endogenous and soluble FL-Ang1. Here again, the Ang2-BD bi-specific variants were found to be more potent antagonists than Ang2-BD_WT_ and cRGD (0.5 μM), with Akt phosphorylation intensity being decreased by 53%, 60%, and 24% for Ang2-BD_BC5_, Ang2-BD_BC6_, and Ang2-BD_BC10_, respectively, and only 4% and 10% for Ang2-BD_WT_ and cRGD, respectively (Fig. 4e, f). To test the downstream signaling of integrin, we evaluated the phosphorylation levels of FAK in TIME cells in vitronectin-coated plates as above. The results demonstrated that the Ang2-BD bi-specific variants (1 μM) significantly inhibited FAK phosphorylation. Here again, the Ang2-BD bi-specific variants were found to be more potent antagonists than Ang2-BD_WT_ and cRGD (1 μM), with FAK phosphorylation intensity being decreased by 50%, 42%, and 10% for Ang2-BD_BC5_, Ang2-BD_BC6_, and Ang2-BD_BC10_, respectively, and no decrease being seen for Ang2-BD_WT_ or cRGD (Fig. 4g, h).

### Inhibition of capillary tube formation, viability, and invasiveness of endothelial cells by Ang2-BD bi-specific variants

**Fig. 5:**
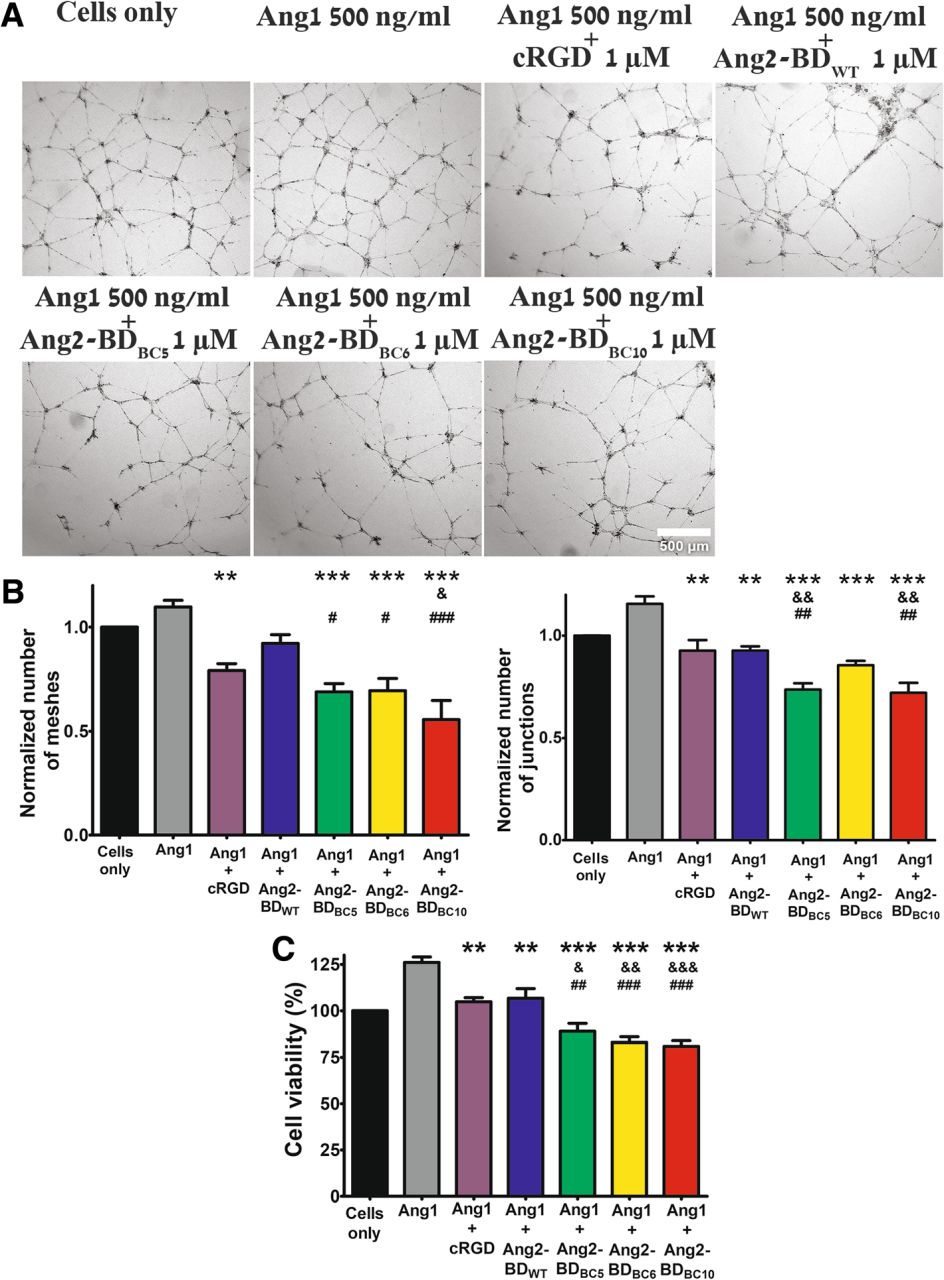
Endothelial cell tube formation and inhibition of cell viability by Ang2-BD bi-specific variants. **a** 3.25 × 10^4^ TIME cells were treated with the indicated proteins overnight and washed, and pictures were taken using a × 2 objective. Scale bar, 500 μm. **b** Images of tube structures were analyzed for the number of generated junctions and the number of meshes in control buffer (cells only; black), 500 ng/ml FL-Ang1 (gray), or a combination of 500 ng/ml FL-Ang1 with 1 μM cRGD (purple), Ang2-BD_WT_ (blue), Ang2-BD_BC5_ (green), Ang2-BD_BC6_ (yellow), or Ang2-BD_BC10_ (red). **c** Cell viability was determined by the XTT assay for control buffer (cells only; black), 500 ng/ml FL-Ang1 (gray), or a combination of 500 ng/ml FL-Ang1 with 2 μM cRGD (purple), Ang2-BD_WT_ (blue), Ang2-BD_BC5_ (green), Ang2-BD_BC6_ (yellow), or Ang2-BD_BC10_ (red). “*” indicates a *P* value < 0.05 upon comparing the results between FL-Ang1 and the tested proteins. “&” indicates a *P* value < 0.05 upon comparing the results between cRGD and the tested proteins. “#” indicates a *P* value < 0.05 upon comparing the results between Ang2-BD_WT_ and the tested proteins (**b**, **c**)

**Fig. 6:**
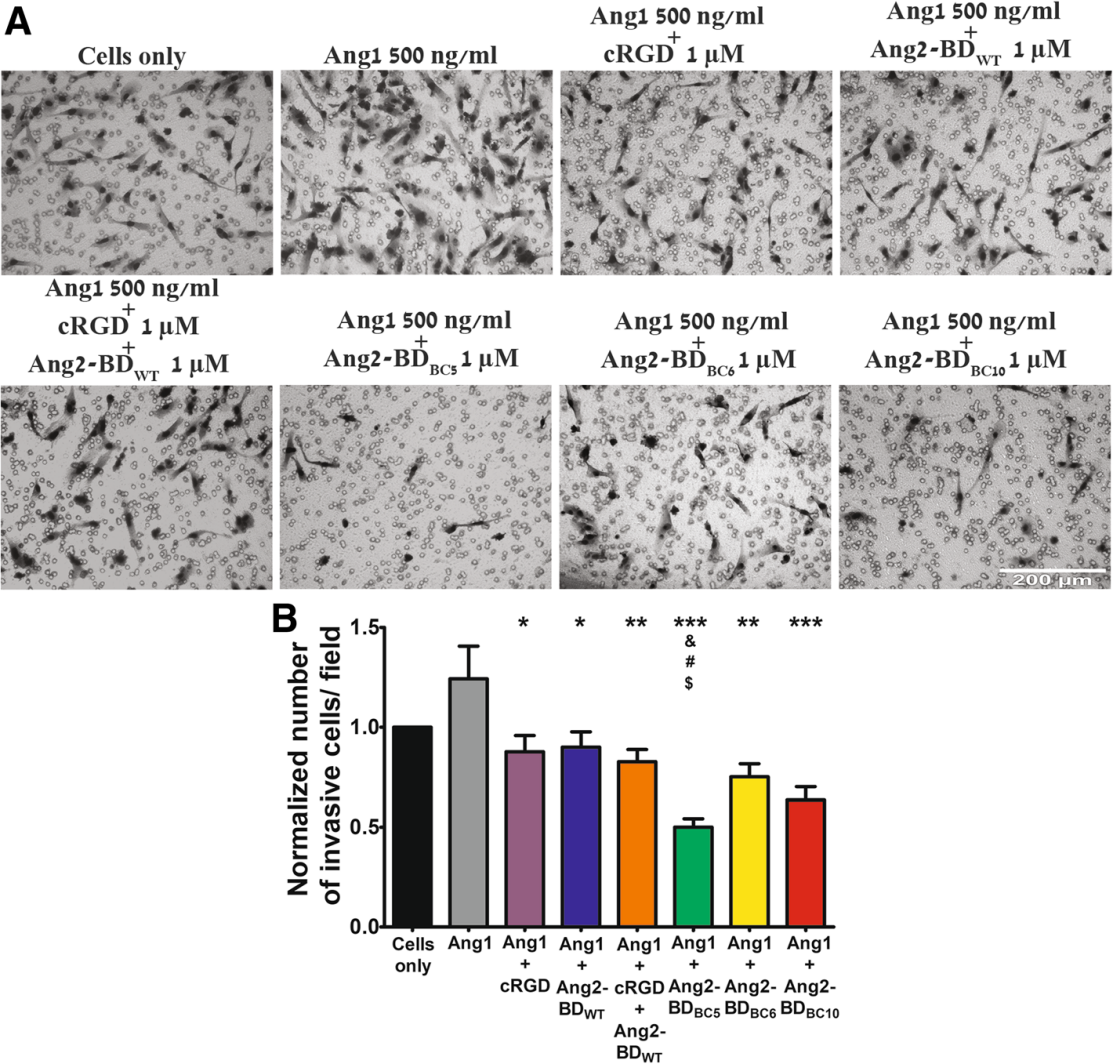
Inhibition of endothelial cell invasiveness by Ang2-BD bi-specific variants. **a** TIME cells were treated with the indicated proteins in Boyden chambers. Scale bar, 200 μm. **b** The invading cells accumulating at the bottom of the membrane were counted in 16 frames for each membrane and analyzed for the number of cells for control buffer (cells only; black), 500 ng/ml FL-Ang1 (gray), or a combination of 500 ng/ml FL-Ang1 with 1 μM cRGD (purple), Ang2-BD_WT_ (blue), cRGD together with Ang2-BD_WT_ (orange), Ang2-BD_BC5_ (green), Ang2-BD_BC6_ (yellow), or Ang2-BD_BC10_ (red). “*” indicates a *P* value < 0.05 upon comparing the results between FL-Ang1 and the tested proteins. “&” indicates a *P* value < 0.05 upon comparing the results between cRGD and the tested proteins. “#” indicates a *P* value < 0.05 upon comparing the results between Ang2-BD_WT_ and the tested proteins. “$” indicates a *P* value < 0.05 upon comparing the results between cRGD + Ang2-BD_WT_ and the tested proteins. The data in Figs. 4, 5, and 6 were analyzed by one-way ANOVA. Data shown represent the average of triplicates from independent experiments, and error bars represent the standard error of the mean

The ability of Ang2-BD bi-specific variants to inhibit capillary tube formation of endothelial TIME cells growing on Matrigel was tested in the presence of FL-Ang1. The Ang2-BD bi-specific variants (1 μM) were superior to the mono-specific proteins Ang2-BD_WT_ and cRGD in inhibiting capillary tube formation, as could be seen by the decrease in the numbers of tubular meshes and tubular junctions (Table 4; Fig. 5a, b). The Ang2-BD bi-specific variants (2 μM and 1 μM, respectively) were also found to decrease endothelial cell viability (Table 4; Fig. 5c) and to exhibit superior potency in inhibiting cell invasiveness, in comparison with the mono-specific controls and their combination (Table 4; Fig. 6a, b).

**Table 4:** Inhibition percentage of capillary tube formation, viability, and invasiveness of endothelial TIME cells growing on Matrigel vs FL-Ang1-treated controls

Inhibitor	Inhibition of tube formation*		Reduction in endothelial cell viability**	Inhibition of invasiveness*
	Reduction in no. of tubular meshes	Reduction in no. of tubular junctions		
Bi-specific				
Ang2-BD_BC5_	38	38	30	60
Ang2-BD_BC6_	37	26	34	40
Ang2-BD_BC10_	50	38	36	49
Mono-specific				
Ang2-BD_WT_	16	20	15	28
cRGD peptide	28	20	17	30

*Inhibitor concentration 1 μM

**Inhibitor concentration 2 μM

## Discussion

Research on Tie2 inhibitors is still an emerging field, despite the plethora of work on human RTKs, in general. The particular challenge in developing specific Tie2 inhibitors derives from off-target effects and toxicity [71]. The very few pre-clinical trial candidates targeting the Ang–Tie2 axis are either antibodies (or peptide mimetics) that target the Ang ligand [72–76] or small molecules that target the Tie2 kinase domain [77]. Neither approach constitutes a viable strategy for specifically targeting Ang–Tie2 interactions as both could lead to unwanted effects. In the first case, Tie2 inhibition could be mediated by inhibition of either Ang1 or Ang2 separately or both together, while in the second case, multi-kinase small molecule inhibitors show low target specificity. In contrast, engineering a natural ligand to function as an antagonist represents a highly effective strategy for creating protein-based biological inhibitors. This strategy is particularly suitable for tailoring specificity into a given protein–protein interaction, because ligands, unlike antibodies, naturally bind the desired epitopes. Our team has recently employed a combinatorial engineering approach to transform the Ang2-BD into a highly potent Tie2 inhibitor with enhanced anti-angiogenic and anti-invasive cellular activities against endothelial cells [54]. Nevertheless, a potential risk inherent in the therapeutic targeting of Tie2 (and of all other RTK multi-families) is that these enzyme-linked receptors do not work in isolation but rather as part of complex enzymatic cascades, in which each RTK may cross-activate other molecules, such as integrins. In that case, the overexpression of integrins could compensate for the loss of Tie2 function. To circumvent this potential limitation, we developed Ang2-BD-based bi-specific inhibitors that simultaneously target both Tie2 and its immediate in vivo target α_v_β_3_ integrin.

In doing so, we demonstrated that Ang2-BD is an excellent scaffold for engineering bi-specific ligands with affinity and specificity for both Tie2 and α_v_β_3_ integrin. To deal with the problem that the RGD sequence in ECM ligands (usually found in the flexible solvent-exposed loops of fibronectin, vitronectin, fibrinogen, and osteopontin [78]) is recognized by many ligands, we generated a mutant XXXRGDXXX loop library, in which the XXXRGDXXX sequence replaced residues 301–308 of Ang2-BD (TFPNSTEE) yet still remained accessible, as shown by our docking results (Fig. 3). In such configuration, Ang2-BD is able to interact with α_v_β_3_ integrin without disrupting the contacts with the native Tie2 receptor, as this receptor interacts with the residues located on the opposite side of the Ang2-BD ligand. In all our library-isolated clones, the RGD motif was found in the center of the loop; no consensus among the flanking residues was noted.

All-atom MD simulations allowed us to propose a model of the Ang2-BD_BC5_–α_v_β_3_ integrin complex and identify contact points between the ligand and receptor. Some contacts are identical to those previously described for the crystal structure of α_v_β_3_ integrin with the RGD ligand [51], e.g., a hydrogen bond between the Asp of RGD and Asn215 of the integrin β_3_ subunit. Other interactions are similar in nature and closely located to those resolved in the crystal, e.g., there is an Arg304–Asp217 salt bridge that “replaces” the Arg304–Asp218 bond in the crystal structure. Overall, the spatial orientation of RGD in our simulations is similar to that in the crystal structure [51] in which Arg and Asp side chains point in opposite directions, making contact with the α_v_ and β_3_ subunits, respectively. Indeed, in the simulations, the Arg side chain was located close to both α_v_ and β_3_ residues, although Arg failed to form a close and stable bond with the α_v_ subunit. It has been shown that Arg adopts various conformations during the series of conformational changes that α_IIb_β_3_ integrin undergoes upon RGD binding [52]. The same work also proposed that Asp serves as a key factor in RGD binding to integrins, while the orientation of Arg could be disordered and flexible. Indeed, the interactions of Asp with β_3_ in our simulations were almost identical to those described in related crystal structures [51, 52].

In light of the above considerations, we propose a putative mode of interaction between the Ang2-BD-based variants and the headpiece of α_v_β_3_ integrin, in which our bi-specific inhibitors can structurally bind both Tie2 and α_v_β_3_ receptors and hence act as antagonists. This proposed mode of action is supported by the following observations. SPR and cell surface staining showed that the three bi-specific variants simultaneously bind both receptors (Fig. 2a). Moreover, the experiments with endothelial cells demonstrated the superior affinity of the bi-specific variants vis-à-vis mono-specific controls (Fig. 2b). Finally, the competition binding experiment showed a partial reduction in the binding of the bi-specific proteins upon the addition of mono-specific inhibitors (Fig. 2c). The three bi-specific proteins, each presenting different amino acids flanking the RGD motif, had different binding affinities for Tie2 and α_v_β_3_ integrin. Selective integrin binding was further achieved as the three proteins displayed high affinity to α_v_β_3_ integrin; moderate affinity to α_v_β_5_, α_5_β_1_, α_v_β_1_, α_v_β_6_, and α_v_β_8_ integrins; and no detectable affinity to α_3_β_1_, α_IIb_β_3_, and α_4_β_7_ integrins (Additional file 1: Figure S2; Table 3).

In correlation with the cell binding results, the three bi-specific Ang2-BD variants presented enhanced inhibitory capabilities, as compared to Ang2-BD_WT_ and cRGD, again highlighting the effects of the dual functionality engineered into these proteins. It had previously been shown that the Tie2–α_5_β_1_ integrin association was significantly enhanced in the presence of an ECM component (collagen or fibrinogen) and fibronectin, the ligand of α_5_β_1_ and α_v_β_3_ integrins [32]. It has also been demonstrated that cooperative Tie2–α_v_β_3_ integrin (and Tie2–α_5_β_1_ integrin) interactions selectively stimulated ERK/MAPK and (PI3-K)/Akt signaling by endothelial cells [32, 34] in the presence of Ang1 and fibronectin, respectively [35]. In agreement with the above, the bi-specific Ang2-BD variants were better inhibitors (relative to Ang2-BD_WT_ and cRGD) of endothelial cell adhesion (Fig. 4a, b); Tie2, Akt, and FAK phosphorylation (Fig. 4c–h); tube formation (Fig. 5a, b); endothelial cell proliferation (Fig. 5c); and invasiveness (Fig. 6a, b) in the presence of Ang1 and vitronectin, the natural ligands of Tie2 and α_v_β_3_ integrin, respectively.

We also showed that the bi-specific proteins were better than their mono-specific counterpart Ang2-BD_WT_ (solely a Tie2 binder) in terms of their ability to inhibit Tie2 receptor phosphorylation, and endothelial tube formation, proliferation, and cell invasion, despite having Tie2 binding affinities similar to or lower than those of Ang2-BD_WT_ (Table 2). This finding is in agreement with the major role of integrins in receptor phosphorylation, endothelial tube formation, and cell invasion. The finding that the bi-specific proteins were also better inhibitors than cRGD (solely an integrin binder) illustrates the distinct contribution of Tie2 to inhibiting these functions. The fact that the bi-specific proteins but not the mono-specific controls exhibited strong antagonistic activity that reduced the levels of receptor phosphorylation, endothelial tube formation, and cell invasion below basal levels suggests that the engineered proteins inhibit both exogenous FL-Ang1 and endogenous Ang1.

This study—the first describing a non-immunoglobulin single domain protein inhibitor targeting most of the known physiological outcomes of Tie2–α_v_β_3_ integrin crosstalk [79]—paves the way for exploiting combinatorial engineering of ligand–Tie2 and ligand–α_v_β_3_ integrin receptor interactions. The novel proteins targeting the Tie2–α_v_β_3_ integrin axis could find clinical application as therapeutics or imaging agents and could also constitute new tools for studying molecular mechanisms and cell signaling pathways involving Tie2 and α_v_β_3_ integrin, which mediate cancer metastasis, angiogenesis, and other important biological processes.

## Conclusions

In summary, this study made two specific breakthrough achievements. (i) We developed, for the first time, non-immunoglobulin multi-specific single domain inhibitors that target all known cancer-promoting functions resulting from Tie2/integrin α_v_β_3_ crosstalk, and (ii) these new inhibitors allowed us to separately explore the role of each signaling molecule (i.e., Tie2 and integrin α_v_β_3_) in cancer cell invasion and angiogenesis.

## Methods

### Preparation of yeast surface display (YSD) Ang2-BD constructs and RGD loop library

The construct for Ang2-BD_WT_ (amino acids 281 to 496) in the pIDT plasmid was obtained by custom gene synthesis (Integrated DNA Technologies). Amplification of the gene was performed using primers containing NheI and BamHI restriction sites at the 3′ and 5′ ends, respectively. The amplified gene was then introduced into the pCTCON yeast display vector (a generous gift from Dane Wittrup, MIT). The pCTCON vector introduces a cMyc epitope at the C-terminus of the encoded protein, allowing for the detection of expression by antibodies. A loop on the Ang2-BD_WT_ construct between residues 301–308 was chosen for library construction. The library was prepared using the NNS degenerate codons, where N = A, C, T, or G and S = C or G. The loop library was constructed with an RGD sequence flanked by three random residues on each side of the RGD motif (GenScript) and homologous recombination into *Saccharomyces cerevisiae* EBY100 cells, as previously described [55]. Library size was approximately 1 × 10^7^ transformants, as estimated by the dilution plating on a selective SDCAA medium (2% dextrose, 1.47% sodium citrate, 0.429% citric acid monohydrate, 0.67% yeast nitrogen base, and 0.5% casamino acids, pH 4.5).

### Screening of YSD Ang2-BD_RGD_ libraries

Yeast-displayed Ang2-BD RGD loop libraries were grown in a selective medium and induced for expression with 2% (*w*/*v*) galactose at 30 °C overnight until OD_600_ = 10.0, according to the established protocols [55]. The library was subjected to five rounds of screening using high-throughput flow cytometric sorting to isolate clones with high affinity for recombinant α_v_β_3_ integrin [human integrin α_v_ subunit (Phe31-Val992), human integrin β_3_ subunit (Gly27-Asp718); R&D Systems]. Library screening was performed using decreasing concentrations of α_v_β_3_ integrin (250 nM, 100 nM, 30 nM, and 10 nM) in sorts 2, 3, 4, and 5, respectively; sort 1 was for positive expression, and Tie2 binding was conducted with 100 nM of α_v_β_3_ integrin. A diagonal sorting gate including 1% of the entire yeast pull was used to select Ang2-BD mutants that bind strongly to α_v_β_3_ integrin. The diagonal sorting gate normalized the binding signal to the amount of protein expressed on the yeast surface. For each round of sorting, yeast cells at an amount of approximately ten times the library size were labeled with solubilized α_v_β_3_ integrin (R&D Systems) and a 1:200 dilution of chicken anti-cMyc antibodies (Thermo Fisher Scientific, Cat# A-21281, RRID:AB_2535826) in integrin-binding buffer [IBB, 20 mM Tris-HCl, pH 7.5, 100 mM NaCl, 1 mM MnCl_2_, 2 mM CaCl_2_, and 1% bovine serum albumin (BSA)] for 1 h at room temperature to facilitate fluorescent detection by flow cytometry. Cells were washed and resuspended in ice-cold PBSA (phosphate-buffered saline with 1% BSA) containing a 1:25 dilution of fluorescein isothiocyanate (FITC)-labeled mouse anti-α_v_ integrin (BioLegend, Cat# 327907, RRID:AB_940558) and a 1:100 dilution of phycoerythrin (PE)-conjugated anti-chicken IgY (Santa Cruz Biotechnology, Cat# sc-3748, RRID:AB_634859). After 25 min of incubation on ice, yeast cells were washed in PBSA and sorted using an iCyt Synergy FACS (fluorescence-activated cell sorting) apparatus [Proteomics Unit, National Institute for Biotechnology in the Negev (NIBN), Ben-Gurion University of the Negev (BGU)]. Sixty isolated clones from the two final sorts were sequenced by extraction of plasmid DNA from the yeast clones using a Zymoprep kit (Zymo Research) and transformed into electrocompetent *Escherichia coli* cells for plasmid miniprep isolation (RBC Bioscience Corp, Taiwan) and DNA sequencing (DNA Microarray and Sequencing Unit, NIBN, BGU). Cells expressing these clones were evaluated for their binding affinity towards α_v_β_3_ integrin by dividing the mean fluorescence intensity (MFI) of the α_v_β_3_ integrin-binding signal by the MFI reflecting expression levels. Binding and expression were detected using anti-α_v_ integrin and anti-cMyc antibodies, respectively. The isolated clones were evaluated for their binding affinity towards Tie2-Fc (R&D Systems) by dividing the MFI of the Tie2 binding signal by the MFI reflecting expression levels. The values obtained were normalized to those obtained with Ang2-BD_WT_. Of the 60 isolated clones, 5 with the highest affinity for α_v_β_3_ integrin and Tie2 were selected.

### Integrin-binding specificity assay

Flow cytometry analysis of 1 × 10^6^ cells of each of the five isolated clones from the RGD loop library was conducted using a 1:200 dilution of chicken anti-cMyc antibody (Thermo Fisher Scientific, Cat# A-21281, RRID:AB_2535826); 50 nM of solubilized α_v_β_3_, α_v_β_5_, α_5_β_1_, α_3_β_1_, α_4_β_7_, or α_IIb_β_3_ integrins (R&D Systems); and 20 nM of soluble Tie2-Fc (R&D Systems) in parallel for 1 h at room temperature. Cells were washed and resuspended in ice-cold PBSA containing a 1:25 dilution of FITC-labeled mouse anti-α_v_/α_5_ integrin (BioLegend, Cat# 327907, RRID:AB_940558, BioLegend Cat# 328308, RRID:AB_2129084), a 1:25 dilution of allophycocyanin (APC)-labeled mouse anti-α_3_/α_4_/α_2b_ integrin (BioLegend, Cat# 343807, RRID:AB_10641703, Cat# 304307, RRID:AB_314433, Cat# 303709, RRID:AB_2129464), and a 1:100 dilution of PE-conjugated anti-chicken IgY (Santa Cruz Biotechnology, Cat# sc-3748, RRID:AB_634859). After 25 min on ice, yeast cells were washed in PBSA and analyzed using BD Accuri C6 flow cytometer (BD Biosciences). These clones (Ang2-BD_WT_, Ang2-BD_BC5_, Ang2-BD_BC6_, Ang2-BD_BC10_, Ang2-BD_BC14_, and Ang2-BD_BC35_) were evaluated for their binding affinity towards α_v_β_3_, α_v_β_5_, α_5_β_1_, α_3_β_1_, α_4_β_7_, and α_IIb_β_3_ integrins by dividing the MFI of the α_v_β_3_ integrin-binding signal by the MFI reflecting expression levels.

### Purification of soluble Ang2-BD proteins

The Multi-Copy *Pichia* Expression Kit (Invitrogen K1750-01) was used to produce the soluble Ang2-BD_RGD_ and Ang2-BD_WT_ protein variants, as previously described [54]. Ang2-BD_RGD_ variants were purified from yeast culture supernatants by metal-chelating chromatography using a 5-ml HisTrap FF column (GE Healthcare) equilibrated with 10 mM imidazole and eluted with 500 mM imidazole. Eluted protein fractions were concentrated and buffer exchanged with 20 mM Hepes, 150 mM NaCl, and pH 7.2 buffer using a 5-kDa cutoff Vivaspin concentrator (GE Healthcare). Gel filtration chromatography was performed using a Superdex 200 column (GE Healthcare) equilibrated with 20 mM Hepes, 150 mM NaCl, pH 7.2, and buffer at a flow rate of 0.5 ml/min on an ÄKTA pure instrument (GE Healthcare). Proteins were separated by SDS-PAGE under non-reducing conditions. Concentrations of all the Ang2-BD_RGD_ protein variants were determined by UV-Vis absorbance at 280 nm and an extinction coefficient of 66,500 M^−1^ cm^−1^. The molecular weights of the purified proteins were determined using a MALDI-TOF REFLEX-IV (Bruker) mass spectrometer (Ilse Katz Institute for Nanoscale Science & Technology, BGU).

### Surface plasmon resonance (SPR) experiments

The binding interactions of Tie2 to Ang2*-*BD_WT_, Ang2-BD_BC5_, Ang2-BD_BC6_, and Ang2-BD_BC10_ were analyzed (Proteomics Unit, NIBN, BGU) by SPR using a ProteOn XPR36 instrument (Bio-Rad) as previously described [54]. The binding interactions of α_v_β_3_, α_v_β_5_, α_v_β_1_, α_v_β_6_, α_v_β_8_, α_5_β_1_, α_4_β_7_, and α_3_β_1_ integrins to Ang2-BD_BC5_, Ang2-BD_BC6_, and Ang2-BD_BC10_ with the extracellular domain of recombinant human α_v_β_3_, α_v_β_5_, α_v_β_1_, α_v_β_6_, α_v_β_8_, α_5_β_1_, α_4_β_7_, and α_3_β_1_ integrins were similarly analyzed (R&D Systems). All integrins were immobilized on the surface of a GLC sensor chip (Bio-Rad) using the amine-coupling reagents sulfo-NHS (0.1 M *N*-hydroxysuccinimide) and EDC (0.4 M 1-ethyl-3-(3-dimethylaminopropyl)-carbodiimide; Bio-Rad). α_v_β_3_, α_v_β_5_, α_v_β_1_, α_v_β_6_, α_v_β_8_, α_5_β_1_, α_4_β_7_, and α_3_β_1_ integrins (5.6 μg) in 10 mM sodium acetate, pH 4.0, were allowed to flow over the activated surfaces of the GLC sensor chip channel at a flow rate of 30 μl/min until target immobilization levels (4300, 7800, 5400, 4600, 5900, 3400, 7100, and 4200 RU, respectively) were reached. BSA (3 μg) in 10 mM sodium acetate, pH 4.5, was then allowed to flow over the activated surfaces of a control GLC sensor chip channel six at a flow rate of 30 μl/min until the target immobilization level (3000 RU) was reached. After protein immobilization, the chip surface was treated with 1 M ethanolamine-HCl at pH 8.5 to deactivate any excess reactive esters. All binding experiments were performed at 25 °C in degassed IBB. Since no suitable regeneration conditions were found for the surface with immobilized α_v_β_3_ integrin, a separate channel was used to test the binding of each Ang2-BD protein. To determine α_v_β_3_-integrin binding interactions, 12.5 to 200 nM of Ang2-BD variants were used, while for α_v_β_5_, α_v_β_1_, α_v_β_6_, α_v_β_8_, α_5_β_1_, α_4_β_7_, and α_3_β_1_ integrins, 1 μM of the Ang2-BD variants was used. For α_v_β_3_ integrin binding, the protein analytes were allowed to flow over the surface-immobilized integrins for 600 s at a flow rate of 30 μl/min, and binding interactions were monitored. Following association, dissociation of the various ligand–receptor complexes was monitored for 400 s. For binding other integrins, the protein analytes were allowed to flow over the surface-immobilized integrins for 400 s at a flow rate of 30 μl/min, the and interactions were monitored. Following association, dissociation of the various ligand–receptor complexes was monitored for 600 s. Each analyte sensorgram run was normalized by subtracting the BSA channel (channel six) run and the zero analyte concentration run. Sensorgram data for α_v_β_3_ integrin binding for all of the Ang2-BD bi-specific variants were analyzed using the 1:1 L model for binding kinetics evaluation and kinetic parameters. Ang2-BD_BC5_ binding to α_v_β_5_, α_v_β_1_, α_v_β_6_, and α_v_β_8_ was analyzed as above. In brief, α_v_β_5_, α_v_β_1_, α_v_β_6_, and α_v_β_8_ integrins (5.6 μg) in 10 mM sodium acetate, pH 4.0, were allowed to flow over the activated surfaces of the GLC sensor chip channel at a flow rate of 30 μl/min until the target immobilization levels (6400, 6300, 4400, and 6100 RU, respectively) were reached. To determine the integrin binding, 62.5 to 1000 nM of Ang2-BD_BC5_ was used. Ang2-BD_BC5_ was allowed to flow over the surface-immobilized integrins for 800 s at a flow rate of 30 μl/min, and the interactions were monitored. Following association, dissociation of the various ligand-receptor complexes was monitored for 700 s. SPR sensorgram curves were fitted into a two-state binding model.

### Dual receptor binding experiments

A ProteOn GLC sensor chip was prepared as described above with immobilized α_v_β_3_ integrin extracellular domain (R&D Systems). α_v_β_3_ integrin (5.6 μg) in 10 mM sodium acetate, pH 4.0, was allowed to flow over the activated surfaces of the GLC sensor chip channel at a flow rate of 30 μl/min until an immobilization level of 4400 RU was reached. Experiments were performed at 25 °C in degassed IBB. Ang2*-*BD_WT_, Ang2-BD_BC5_, Ang2-BD_BC6_, or Ang2-BD_BC10_ (at a concentration of 400 nM) was allowed to flow over the integrin-immobilized surface for 400 s at a flow rate of 30 μl/min. Thereafter, the extracellular domain of recombinant human Tie2 (rhTie2), also at 400 nM, was allowed to flow over the surface for 150 s. Dissociation of the complex was monitored for 650 s. Injection of running buffer followed by rhTie2 in IBB served as negative control.

### Cell-binding assays

Human telomerase-immortalized microvascular endothelium (TIME) cells (ATCC, Cat# CRL-4025, RRID:CVCL_0047) were cultured in growth-factor-depleted Vascular Cell Basal Medium (ATCC) supplemented with 2% fetal bovine serum (FBS) and growth factor supplements (ATCC). For binding assays, 10^5^ cells were suspended and incubated with different concentrations of Ang2-BD variants in a total volume of 200 μl PBSA, followed by incubation at 4 °C for 2 h with gentle agitation. Cell suspensions were centrifuged at 150*g* at 4 °C for 5 min and washed with 100 μl PBSA, followed by centrifugation at 150*g* at 4 °C for 5 min twice more. Cells were then resuspended in 100 μl PBSA containing a 1:200 dilution of APC-conjugated anti-FLAG antibodies (BioLegend, Cat# 637308, RRID:AB_2561497). After 30 min on ice, the cells were washed twice in PBSA and analyzed by flow cytometry with a BD Accuri C6 flow cytometer (BD Biosciences). Mean fluorescence values were generated using FlowJo software (Treestar). For the competitive binding assay, cells were treated as described above with added full-length human Ang1 (FL-Ang1), cRGD peptide, or a combination of the two. Since FL-Ang1 exists in different oligomeric states, the FL-Ang1 concentration is reported in this work as mass concentration units instead of molar concentration units. MFI was detected using PE-conjugated anti-FLAG antibody (BioLegend, Cat# 637309, RRID:AB_2563147) and analyzed by flow cytometry with a BD Accuri C6 flow cytometer. For receptor level detection, 10^5^ cells were harvested, resuspended in 100 μl PBSA with 1:100 Alexa Fluor 647-labeled anti-human Tie2 antibodies (BioLegend, Cat# 334210, RRID:AB_2203206) or (FITC)-labeled anti-human α_v_β_3_ integrin antibodies (Millipore, Cat# MAB1976F, RRID:AB_94482), incubated at 4 °C for 30 min, and then analyzed by flow cytometry.

### Docking modeling and simulation of α_v_β_3_ and Ang2-BD_BC5_ complex

Molecular coordinates of the α_v_β_3_ binding domains were taken from the 1L5G PDB structure [51] (residues 1–438 of the α_v_ subunit and 55–432 of the β_3_ subunit). The coordinates of the binding domain of Ang2 were obtained from the 1Z3S PDB structure [56] (residues 280–495). The Ang2-BD_BC5_ mutant was created by replacing residues 301–308 of the native protein with residues NTCRGDCLP using the PyMOL Molecular Graphics System, Version 1.8 (De Lano). Each structure was energy-minimized using the Gromacs 4.6.7 package of programs [57], and the receptor–ligand docking procedure was performed by a PatchDock server [58]. To avoid irrelevant structures, potential binding sites both for the receptor and the ligand were defined according to PatchDock recommendations. Slight variations in the interaction restraints yielded a total of 421 structures. Docking solutions were clustered with a 0.6-nm cutoff using Gromacs. The most prominent cluster (41% of total) was subjected to molecular dynamics (MD) simulation with Gromacs 4.6.7. Two identical simulations were carried out with the GROMOS 53a6 force field [59], yielding similar results. The protein was immersed in a dodecahedral box, filled with simple point charge (SPC) [60] water molecules and ions that extended to at least 1.2 nm from the edge of the protein. The whole system was subjected to energy minimization using the steepest descent algorithm until the force component of the system was smaller than 1000 kJ mol^−1^ nm^−1^. Equilibration with the solvent was initiated by a 40 ps position-restrained simulation under a constant force of 1000 kJ mol^−1^ nm^−1^ at 300 K. Next, the system was simulated without restraints for 3 ns, allowing for equilibration. The final structure was used for a 10-ns MD simulation as detailed below.

MD simulations were run under NPT (constant number of particles, pressure, and temperature) conditions, relying on Berendsen’s coupling algorithm for maintaining constant temperature and pressure (*P* = 1 bar, τp = 0.5 ps, *T* = 300 K, τR = 0.1 ps) [61]. A LINCS (linear constraint solver) algorithm [62] was used to constrain the lengths of all bonds; the water molecules were restrained by the SETTLE algorithm. Long-range electrostatic interactions were treated by the particle mesh Ewald method [63]. Distances and electrostatic and Lennard–Jones potentials were analyzed with the tools provided by the GROMACS package, while snapshots were prepared by the VMD program [64].

The Tie2 structure was obtained from the 2GY7 PDB structure (residues 23–445), and its coordinates were aligned to a simulated Ang2-BD_BC5_–α_v_β_3_ complex to show the possibility of simultaneous binding of Ang2-BD_BC5_ to α_v_β_3_ integrin and Tie2.

### Cell adhesion assays

Inhibition of adhesion of TIME cells to vitronectin was determined in 96-well microplates coated with human vitronectin (R&D Systems). Ang2*-*BD_WT_, Ang2-BD_BC5_, Ang2-BD_BC6_, Ang2-BD_BC10_, or cRGD peptide (Merck Millipore) (1 μM) was mixed with 5 × 10^4^ TIME cells and plated on vitronectin-coated wells either with or without 500 ng/ml of FL-Ang1, incubated at 37 °C/5% CO_2_ for 2 h, and washed twice with PBS. A solution of 0.2% crystal violet in 10% ethanol was added to the wells for 10 min, which were then washed three times with PBS. Solubilization buffer (a 1:1 mixture of 0.1 M NaH_2_PO_4_ and ethanol) was added, and the plate was shaken gently for 15 min. Absorbance was measured at 600 nm using a microtiter plate reader (BioTek Instruments). Background signals generated with a negative control containing no cells were subtracted from the data.

### Tie2, Akt, and FAK phosphorylation assays

Confluent TIME cells were cultured in growth-factor-depleted Vascular Cell Basal Medium supplemented with 0.5% FBS for 12 h at 37 °C/5% CO_2_ on human vitronectin-coated 12-well plates prior to experimentation. The cells were then washed with PBS, and the medium was exchanged with fresh Vascular Cell Basal Medium-depleted of growth factors and serum. After pre-treatment with 1 mM sodium orthovanadate (Na_3_VO_4_; Sigma) for 15 min, the cells were co-incubated for 15 min for Tie2 and FAK and for 30 min for Akt at 37 °C with either commercial full-length rhAng1 as positive control (R&D Systems) or a combination of full-length rhAng1 and the Ang2-BD bi-specific variants. Non-stimulated cells served as negative control. The cells were then washed twice with PBS plus 1 mM Na_3_VO_4_ and lysed in ice-cold lysis buffer [20 mM HEPES, pH 7.4, 150 mM NaCl, 1% TritonX-100, 1 mM Na_3_VO_4_, and 1× complete protease inhibitor cocktail tablet (Roche)]. The cells were scraped from the culture plate wells, and the lysates were clarified by centrifugation (13,000 rpm for 30 min at 4 °C). Protein concentration was measured by the BCA assay (Thermo Fisher Scientific), and equivalent amounts of each lysate sample were analyzed by duplicate 10% SDS-PAGE and transferred to duplicate PVDF membranes (BioRad). Blots were blocked (5% BSA, 50 mM Tris-HCl, pH 7.4, 150 mM NaCl, 0.1% Tween 20) for 1 h at room temperature and probed with 1:500 dilution anti-phospho-Tie2-specific rabbit polyclonal (R&D Systems, Cat# AF2720, RRID:AB_442172) and 1:1000 dilution anti-Tie2-specific rabbit monoclonal antibodies (Cell Signaling Technology, Cat# 7403S, RRID:AB_10949315), 1:1000 dilution anti-phospho-Akt-specific (Cell Signaling Technology, Cat# 4060, RRID:AB_2315049), and 1:1000 dilution anti-Akt-specific antibodies (Cell Signaling Technology, Cat# 4691, RRID:AB_915783) or 1:1000 dilution anti-phospho-FAK-specific (Cell Signaling Technology, Cat# 8556S, RRID:AB_10891442) and 1:1000 dilution anti-FAK-specific antibodies (Cell Signaling Technology, Cat# 3285S, RRID:AB_10694068) overnight at 4 °C. Membranes were washed three times with TBST (50 mM Tris-HCl, pH 7.4, 150 mM NaCl, 0.1% Tween 20) and probed with 1:1000 dilutions HRP-linked anti-rabbit antibodies (Cell Signaling Technology, Cat# 7074, RRID:AB_2099233) for 1 h at room temperature. Membranes were washed three times with TBST and then visualized and quantified using chemiluminescence (ECL, Biological Industries) and ImageJ software, respectively. The intensities of the phospho-Tie2, phospho-Akt, and phospho-FAK bands were adjusted for the expression of total Tie2, Akt, and FAK for each experiment. Blots were stripped and re-probed with 1:1000 dilution anti-β-actin antibodies (Cell Signaling Technology, Cat# 4970, RRID:AB_2223172) for further normalization. Each condition was repeated in triplicate. Phosphorylated protein (Tie2, Akt, and FAK) band intensities (as measured by ImageJ software) were normalized to the respective total protein levels, and this value was subsequently normalized to the total amount of β-actin for each sample. For each condition, a representative band is shown.

### Matrigel endothelial tube formation assay

Serum-reduced Matrigel (10 mg/ml; BD Biosciences) was thawed overnight at 4 °C, and 150 μl was added to each well of a 48-well microtiter plate and allowed to solidify for 1 h at 37 °C. The wells were incubated with 3.25 × 10^4^ TIME cells plus 500 ng/ml rhAng1 either alone or with 1 μM of Ang2*-*BD_WT_, Ang2-BD_BC5_, Ang2-BD_BC6_, Ang2-BD_BC10_, or cRGD peptide (Merck Millipore). The cells were incubated for 16–18 h at 37 °C/5% CO_2_ and then washed twice in HBSS (Hanks’ balanced salt solution; Sigma). Capillary tube formation was observed using EVOS Cell Imaging Systems microscope (ThermoFisher Scientific). Images were collected with an EVOS × 2 Objective. The total number of meshes and the number of junctions of the tubes were quantified by the analysis of digitized images of the capillary-like structures using ImageJ software and the Angiogenesis Analyzer plugin.

### Cell viability assay

The effects of Ang2-BD bi-specific variants on the growth and survival of TIME cells were assessed by an XTT assay (2,3-bis [2-methoxy-4-nitro-5-sulfophenyl]-2H-tetrazolium-5-carboxanilide inner salt assay; Biological Industries). TIME cells were seeded (7500 cells per well) on a human vitronectin-coated 96-well microplate (R&D Systems) and incubated at 37 °C/5% CO_2_ for 24 h. The medium was then replaced with fresh Vascular Cell Basal Medium supplemented with 2% FBS and growth factor supplements, and the cells were incubated with 500 ng/ml of rhAng1 either alone or with 2 μM Ang2*-*BD_WT_, Ang2-BD_BC5_, Ang2-BD_BC6_, Ang2-BD_BC10_, or cRGD peptide (Merck Millipore). The cells were incubated for 16–18 h at 37 °C/5% CO_2_. Viable cells from each condition were measured by XTT at UV 450 nm, as described in the manufacturer’s protocol. The UV readings of the cell-only control were normalized to 100%, and readings from cells treated with the Ang2-BD variants were expressed as a percentage of the control.

### Invasion assay

An in vitro Boyden chamber assay was performed using ThinCert 24-well inserts (Greiner Bio-One). ThinCert cell culture insert membranes were coated with Matrigel (Corning) diluted in Vascular Cell Basal Medium (ATCC) at a 1:30 ratio. The lower compartment was filled with 600 μl of Vascular Cell Basal Medium supplemented with 2% FBS. TIME cells (2 × 10^4^), with or without Ang2-BD variants and Ang1, were incubated in 200 μl supplement-free Vascular Cell Basal Medium, added to the pre-coated ThinCert cell culture inserts, and incubated for 20 h at 37 °C/5% CO_2_. Invasive cells were stained with a DippKwik stain kit (American MasterTech Scientific) and detected by an EVOS FL Cell Imaging System at × 20 magnification. Quantification was performed by counting 16 fields for each membrane. Analysis of digitized images was performed using ImageJ software and a Cell Colony Edge Analyser.

### Statistical analysis

Data were analyzed with GraphPad Prism version 5.00 for Windows (La Jolla, CA). Data shown in all the figures are the means of triplicate from independent experiments, and error bars represent the standard error of the mean. Statistical significance was determined by column statistics and one-way ANOVA analysis. A *P* value < 0.05 was considered statistically significant.

## Additional files

Additional file 1: **Figures S1–S5**, **Tables S1–S2.** **Figure S1.** Production and purification of soluble Ang2-BD bi-specific variants. **Figure S2.** Surface plasmon resonance (SPR) analysis. **Figure S3.** Expression of Tie2 receptor and α_v_β_3_ integrin by TIME cells. **Figure S4.** RMSD values of MD simulation. **Figure S5.** Distances and energies of interactions between Ang2-BD_BC5_ and α_v_β_3_ integrin. **Table S1.** Interacting Residues between RGD and the β_3_ subunit of α_v_β_3_ integrin. Table S2. Protein sequences of Ang2-BD variants. (DOCX 3584 kb)

Additional file 2: Supporting data values. (XLSX 4536 kb)

## Abbreviations

Ang: Angiopoietin
Ang2-BD: Angiopoietin 2-binding domain
APC: Allophycocyanin
BSA: Bovine serum albumin
FACS: Fluorescence-activated cell sorting
FITC: Fluorescein isothiocyanate
MD: Molecular dynamics
MFI: Mean fluorescence intensity
PE: Phycoerythrin
RTK: Receptor tyrosine kinases
RU: Response unit
SEC: Size-exclusion chromatography
SPR: Surface plasmon resonance
TIME: Telomerase-immortalized human microvascular endothelium
YSD: Yeast surface display
