# Supplementary material for: Targeting the Tie2–αvβ3 integrin axis with bi-specific reagents for the inhibition of angiogenesis
Source: BMC Biol. 2018 Aug 17;16:92. doi: 10.1186/s12915-018-0557-9 (PMC6097439; doi:10.1186/s12915-018-0557-9)
Supplement: Supplementary file 1 — Figures S1–S5, Tables S1–S2. Figure S1. Production and purification of soluble Ang2-BD bi-specific variants. Figure S2. Surface plasmon resonance (SPR) analysis. Figure S3. Expression of Tie2 receptor and αvβ3 integrin by TIME cells. Figure S4. RMSD values of MD simulation. Figure S5. Distances and energies of interactions between Ang2-BDBC5 and αvβ3 integrin. Table S1. Interacting Residues between RGD and the β3 subunit of αvβ3 integrin. Table S2. Protein sequences of Ang2-BD variants. (DOCX 3584 kb) [file 12915_2018_557_MOESM1_ESM.docx]

**Supplementary information: Figures S1-S5, Tables S1-S2**


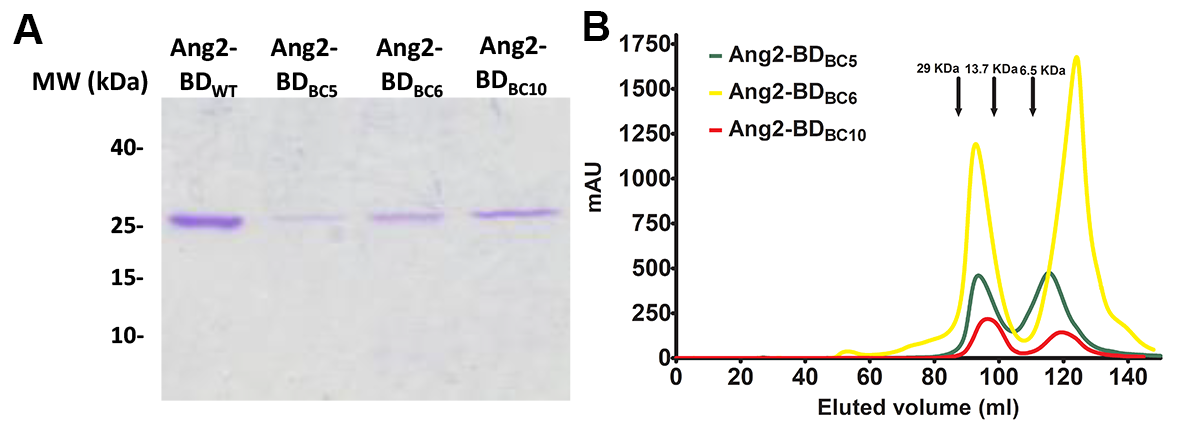


**­**

**Figure S1: Production and purification of soluble Ang2-BD bi-specific variants.**

**(A)** SDS-PAGE analyses of purified Ang2-BD_WT_, Ang2-BD_BC5_, Ang2-BD_BC6_ and Ang2-BD_BC10_. **(B)** Size-exclusion chromatography (SEC) was used to purify Ang2-BD bi-specific variants. Shown is a representative separation of Ang2-BD_BC5_ (green), Ang2-BD_BC6_ (yellow) or Ang2-BD_BC10_ (red) with known protein size standard elution volumes for carbonic anhydrase (29 kDa), ribonuclease A (13.7 kDa) and aprotinin (6.5 kDa).


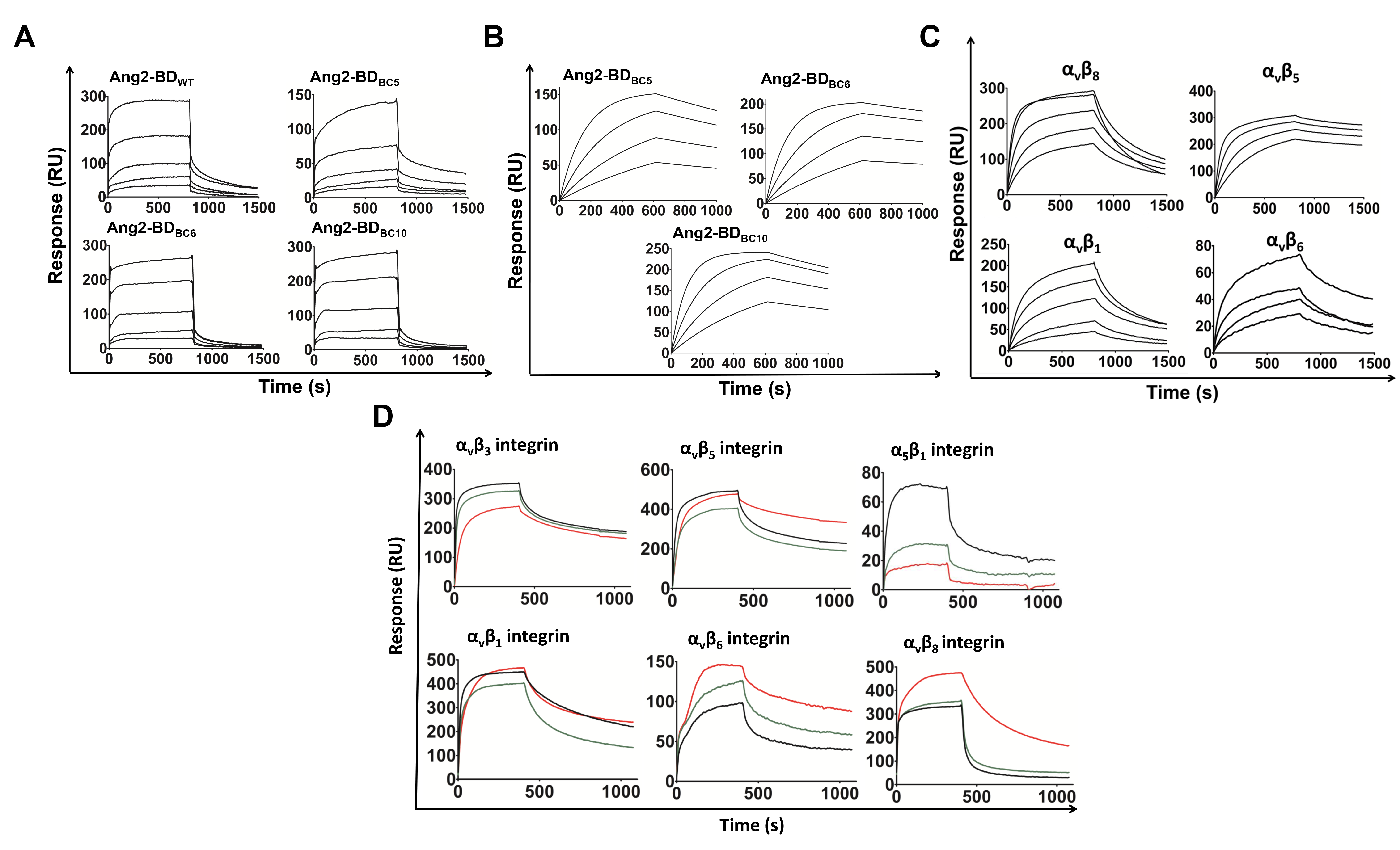


**Figure S2: Surface plasmon resonance (SPR) analysis.**

**(A)** Representative SPR sensorgrams of Ang2-BD_WT_, Ang2-BD_BC5_, Ang2-BD_BC6_ and Ang2-BD_BC10_ (31.25–500 nM) binding to immobilized Tie2. **(B)** Representative SPR sensorgrams of Ang2-BD_BC5_, Ang2-BD_BC6_ and Ang2-BD_BC10_ (12.5–200 nM) binding to immobilized α_v_β_3_ integrin. **(C)** Representative SPR sensorgrams of Ang2-BD_BC5_ (62.5–1000 nM) binding to immobilized α_v_β_8_, α_v_β_5,_ α_v_β_1,_ and α_v_β_6_ integrins. **(D)** Representative SPR sensorgrams of Ang2-BD_BC5_ (red; 1 μM), Ang2-BD_BC6_ (green; 1 μM) and Ang2-BD_BC10_ (black; 1 μM) binding to immobilized α_v_β_3_, α_v_β_5,_ α_5_β_1,_ α_v_β_1,_ α_v_β_6_ and α_v_β_8_ integrins. Binding to α_4_β_7_, α_IIb_β_3_ and α_3_β_1_ integrins (1 μM Ang2-BD variants) was not observed.


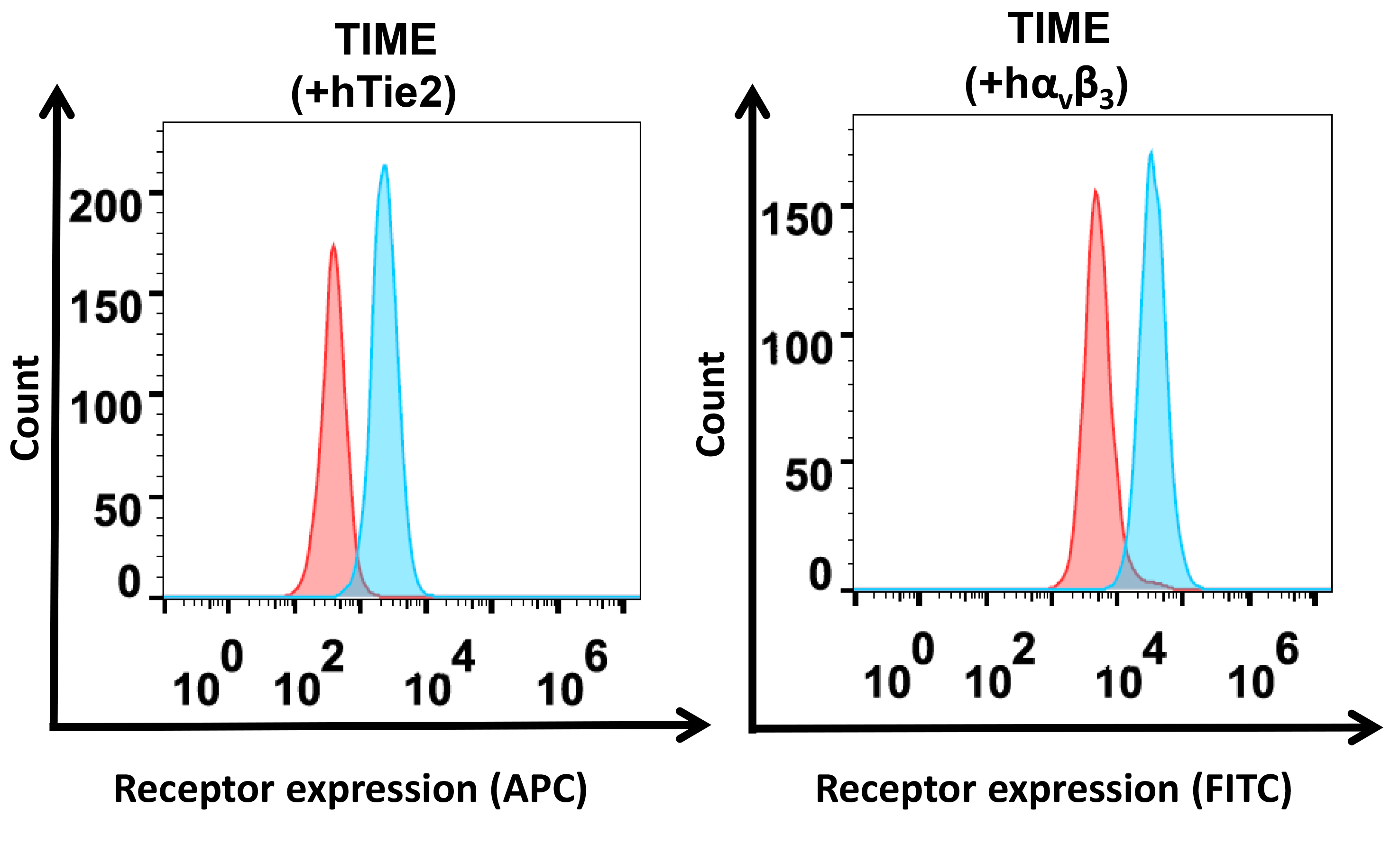


**Figure S3: Expression of Tie2 receptor and α_v_β_3_ integrin on TIME cells.**

**(A)** Cell surface expression of Tie2 (blue) and **(B)** α_v_β_3_ integrin (blue) as opposed to cells only (red). 1×10^5^ TIME cells were stained with APC-labeled anti-human Tie2 antibodies and FITC-labeled anti-human α_v_β_3_ integrin antibodies and incubated at 4°C for 30 min before analysis by flow cytometry.


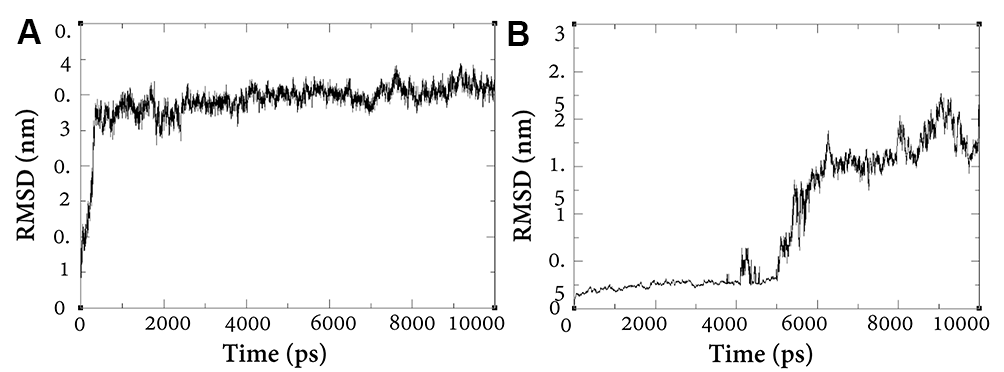


**Figure S4**: **RMSD values of MD simulation.**

**(A)** Calculation of RMSD for the interface between Ang2-BD_BC5_ and α_v_β_3_ integrin. Calculations made specifically for the complex interface showed high stability with a low RMSD value, which was reached as early as 0.5 ns. **(B)** Calculation of RMSD for the whole complex. Note that RMSD values started to rise from 5 ns, and were still relatively unstable at the end of the simulations.


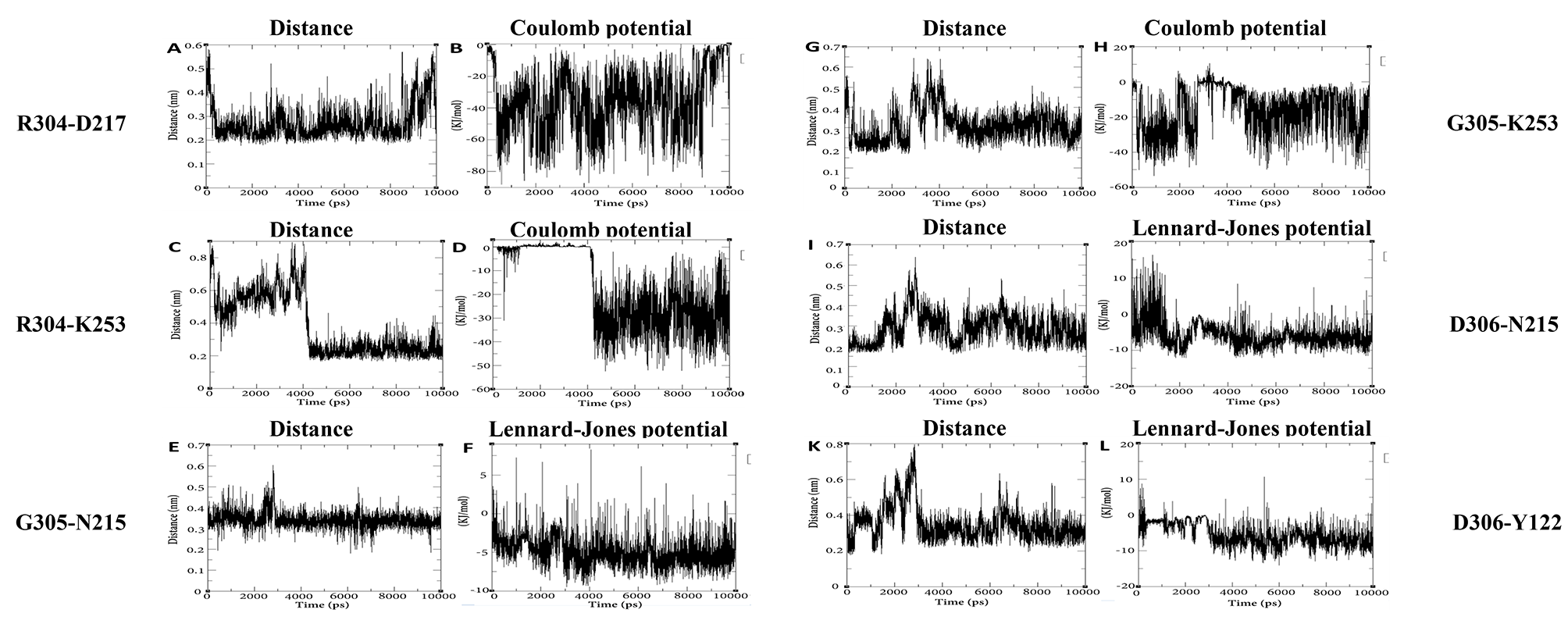


**Figure S5**: **Distances and energies of interactions between Ang2-BD_BC5_ and α_v_β_3_ integrin.**

Minimal distances (in nm) between closest atoms and interaction energies were calculated during a 10 ns MD simulation and presented as a function of simulation time. Electrostatic forces and interaction energies, calculated as Coulomb and Lennard-Jones potentials, respectively, are also presented as a function of simulation time. **(A)** R304-D217 bond distance. **(B)** R304-D217 bond Coulomb potential. **(C)** R304-K253 bond distance. **(D)** R304-K253 Coulomb potential. **(E)** G305-N215 bond distance. **(F)** G305-N215 Lennard-Jones potential. **(G)** G305-K253 bond distance. **(H)** G305-K253 Coulomb potential. **(I)** D306-N215 bond distance. **(J)** D306-N215 Lennard-Jones potential. **(K)** D306-Y122 bond distance. **(L)** D306-Y122 Lennard-Jones potential. The left panels show the distance between the interacting atoms, and the right panels show the energy of the interaction. Electrostatic energy (Coulomb potential) – evident in panels B, D and H – reached values of 30-50 kJ for those time frames in which the distance of 0.2-0.3 nm allowed strong interaction. The highest value for electrostatic energy – observed for the R304-D217 bond (panel B) – may be attributed to an interaction between fully and oppositely charged atoms, creating a strong salt bridge in the complex. In panels F, J and L, it can be seen that the energy of interactions created by uncharged atoms reached values of 5-8 kJ, according to distances of approximately 0.3 nm. These bonds were weaker than those constituting the salt bridge but still contributed to the cumulative effect of the binding of RGD to α_v_β_3_ integrin. In addition to the bonds presented in the figure, several other interactions of RGD were evident (data not shown).

| **Table S1**. Interacting residues between RGD and the β_3_ subunit of α_v_β_3_ integrin | | |
| --- | --- | --- |
| RGD ligand of Ang2-BD_BC5_ | β_3_ residue number | Comments |
| R304 | D217 | Salt bridge between side chains |
|  | K253 | Carbonyl oxygen of R with positively charged K amine group |
| G305 | N215 | Carbonyl oxygen of G with N amine group |
|  | K253 | Carbonyl oxygen of G with positively charged K amine group |
| D306 | N215 | Backbone to backbone hydrogen bond |
|  | Y122 | D carboxylate with Y aromatic side chain |

| **Table S2. Protein sequences of Ang2-BD variants** | |
| --- | --- |
| **Ang2-BD variant** | **Protein sequence** |
| **Ang2-BD_WT_** | **EFRDCAEVFKSGHTTNGIYTLTFPNSTEEIKAYCDMEAGGGGWTIIQ**  **RREDGSVDFQRTWKEYKVGFGNPSGEYWLGNEFVSQLTNQQRYVLK**  **IHLKDWEGNEAYSLYEHFYLSSEELNYRIHLKGLTGTAGKISSISQPGND**  **FSTKDGDNDKCICKCSQMLTGGWWFDACGPSNLNGMYYPQRQNTNKF**  **NGIKWYYWKGSGYSLKATTMMIRPADF** |
| **Ang2-BD_BC5_** | **EFRDCAEVFKSGHTTNGIYTLNTCRGDCLPIKAYCDMEAGGGGWTIIQ**  **RREDGSVDFQRTWKEYKVGFGNPSGEYWLGNEFVSQLTNQQRYVLKIHLK**  **DWEGNEAYSLYEHFYLSSEELNYRIHLKGLTGTAGKISSISQPGND**  **FSTKDGDNDKCICKCSQMLTGGWWFDACGPSNLNGMYYPQRQNTNKF**  **NGIKWYYWKGSGYSLKATTMMIRPADF** |
| **Ang2-BD_BC6_** | **EFRDCAEVFKSGHTTNGIYTLREGRGDNVDIKAYCDMEAGGGGWTIIQ**  **RREDGSVDFQRTWKEYKVGFGNPSGEYWLGNEFVSQLTNQQRYVLKIHLK**  **DWEGNEAYSLYEHFYLSSEELNYRIHLKGLTGTAGKISSISQPGND**  **FSTKDGDNDKCICKCSQMLTGGWWFDACGPSNLNGMYYPQRQNTNKF**  **NGIKWYYWKGSGYSLKATTMMIRPADF** |
| **Ang2-BD_BC10_** | **EFRDCAEVFKSGHTTNGIYTLYPGRGDNPDIKAYCDMEAGGGGWTIIQ**  **RREDGSVDFQRTWKEYKVGFGNPSGEYWLGNEFVSQLTNQQRYVLKIHLK**  **DWEGNEAYSLYEHFYLSSEELNYRIHLKGLTGTAGKISSISQPGND**  **FSTKDGDNDKCICKCSQMLTGGWWFDACGPSNLNGMYYPQRQNTNKF**  **NGIKWYYWKGSGYSLKATTMMIRPADF** |
